# Evolutionary dynamics of enlarged neo-sex chromosomes and novel pseudoautosomal regions in Sylvioidea songbirds

**DOI:** 10.1038/s42003-026-10603-3

**Published:** 2026-07-04

**Authors:** Hanna Sigeman, Simon J. Ellerstrand, Bengt Hansson

**Affiliations:** 1 Department of Biology, Science for Life Laboratory, BECC – Biodiversity and Ecosystem services in a Changing Climate, Lund University, Lund, Sweden; 2 Ecology and Genetics Research Unit, University of Oulu, Oulu, Finland; 3 Department of Medical Biochemistry and Microbiology, Uppsala University, Uppsala, Sweden

**Keywords:** Evolutionary genetics, Evolutionary biology

## Abstract

Recombining regions of sex chromosomes, pseudoautosomal regions (PARs), are essential for proper segregation of the sex chromosome pair during meiosis. Yet, the evolutionary dynamics of PARs remain poorly understood, particularly in relation to chromosomal translocation and fusion events. Using comparative analyses of enlarged neo-sex chromosomes across Sylvioidea songbirds, we show that the ancestrally conserved songbird PAR has independently ceased recombining multiple times, each coinciding with the emergence of novel PAR in newly sex-linked regions. These PAR transitions were associated with lineage-specific fusions, but were driven by subsequent recombination suppression events rather than the fusions themselves. Our data further support hypotheses predicting that recombination suppression expansions are preceded by sex-associated sequence divergence near the PAR boundary. Together, our results reveal remarkable PAR diversity among birds and demonstrate how chromosome fusions relax constraints on existing PARs, facilitating recombination suppression and enabling PAR transitions along evolving sex chromosomes.

## Abstract (web summary)

Comparative genomics of neo-sex chromosomes reveals how chromosome fusions and subsequent recombination suppression drive pseudoautosomal region turnover across Sylvioidea songbirds.

## Introduction

Sex chromosomes originate through the acquisition of one or more sex-determining genes, typically followed by the evolution of recombination suppression in the surrounding genomic region^1–4^. From this point onward, sex chromosomes comprise two primary regions: a non-recombining region (NRR), where mutations drive differentiation between the sex chromosome pair (XY or ZW), and a pseudoautosomal region (PAR), which remains partially sex-linked but continues to recombine in one or both chromosome ends. The PAR plays an essential mechanistic role in ensuring proper pairing and segregation of the sex chromosome pairs during meiosis^5^. It is also a unique genomic domain, retaining sequence homology through recombination, yet differing from autosomes due to its partial linkage with the sex-determining region^5,6^. This linkage increases the likelihood of maintaining sex-associated polymorphisms, such as alleles with opposing fitness effects in the sexes (i.e., sexually antagonistic polymorphism), particularly near the boundary between the PAR and the NRR^5,7,8^. The accumulation of such sexually antagonistic alleles is predicted to favour further expansion of recombination suppression along the sex chromosomes^9–11^, as formulated in the sexual antagonism hypothesis (see ref. ^12^ for additional hypotheses). However, recent models suggest that the conditions under which this hypothesis applies may be more limited than previously assumed^13,14^.

The PAR is evolutionary dynamic. It may contract through recurrent recombination suppression events along the sex chromosomes, sometimes to the extent that only a small recombining fraction remains. Conversely, the PAR can expand via translocations of autosomal material to the sex chromosomes^5^, where subsequent recombination suppression may shift the PAR boundary into the translocated region. Systems in which the PAR has changed in size, location or gene content offer valuable opportunities to investigate the genomic architectures and evolutionary forces that facilitate or constrain the spread of recombination suppression. Comparative analyses of genes located in the PAR, the NRR and autosomes across species can shed light on several poorly understood aspects of PAR biology. These include patterns of gene retention versus absorption into the NRR, the impact of recombination suppression on sex-specific gene expression, and how the PAR responds to sexually antagonistic selection^5,8,14,15^. Notably, the PAR typically displays high recombination rates^16,17^, which enhances the efficacy of selection by reducing linked selection and genetic drift caused by Hill-Robertson interference and related processes^18–20^. Moreover, the PAR is characterised by genome instability and pronounced sequence variation both within and between species^17,21^, which may contribute to hybrid sterility and act as a driver of reproductive isolation^22^. Despite its evolutionary importance, the dynamics of PAR evolution remain poorly understood in most clades (but see, e.g., refs. ^22,23^.), particularly in relation to chromosomal translocation and fusion events (but see, e.g., refs. ^24–26^).

In this study, we investigate the evolutionary dynamics of the PAR in songbirds of the superfamily Sylvioidea (*sensu* refs. ^27,28^), a clade in which five documented sex chromosome–autosome fusion events have translocated substantial chromosomal segments to the ancestral sex chromosomes^29–39^. Some of these translocations are lineages specific, resulting in varying degrees of sex chromosome enlargement across Sylvioidea. One translocation event occurred early in the Sylvioidea radiation, involving a part of chromosome 4A (as designated in songbirds)^29–32^ that merged to the non-PAR end of the Z chromosome and also became physically linked to the W chromosome^31,32^. The remaining four translocations, involving parts of chromosomes 3, 4, 5 and 8, are distributed across different Sylvioidea lineages^33–39^. Neo-sex chromosomes are rare in other avian lineages^40–44^, making these Sylvioidea translocations exceptional relative to the otherwise highly conserved avian sex chromosomes, which originated over 100 million years ago and are largely non-recombining, with the exception of some paleognaths^23,45^. To date, the PAR has been characterised in a few songbird species (Passeriformes), including *Ficedula albicollis*^46^, *Taeniopygia guttata*^47^, and five bird-of-paradise species (family Paradisaeidae)^48^. These PARs range from ~450 to 870 kb in size and exhibit high homology^46–48^. Our objectives were to (i) test whether the unique sex chromosome–autosome fusions in Sylvioidea have led to recombination suppression of the typically conserved ancestral songbird PAR^46–48^; (ii) characterise the structure of these enlarged neo-sex chromosomes by positioning the translocated regions; (iii) identify novel PARs within the translocated chromosomes; and (iv) assess whether genes located closer to the PAR boundary in species retaining the ancestral PAR exhibit increasing sex-associated sequence divergence, as predicted by sex chromosome theory^5,6,8^.

## Results

### Sex-linked chromosomes and location of non-recombining regions (NRRs) in Sylvioidea

Building on previous findings of sex-linked chromosomes in Sylvioidea^32,36–39^, we categorised the 16 species included in the present study (14 Sylvioidea and two non-Sylvioidea species) into seven groups (Group I–VII), each defined by shared NRRs (groupings follow ref. ^39^). The chromosomal positions of each NRR (Supplementary Fig. 1; Supplementary Table 1) were inferred through synteny with the chromosome-level *Taeniopygia* genome assembly taeGut3.2.4^49^, which we had modified by placing the previously unanchored scaffold containing the PAR (scaffold ID: Z_random:0–468 kb) at the beginning of chromosome Z (an adjustment aligning with an updated *Taeniopygia* assembly; GCF_008822105.2; ref. ^50^).

NRRs occur on chromosome Z in all 16 species (Group I–VII), 4A in all 14 Sylvioidea species (Group II–VII), 4 in *Cisticola juncidis* (family Cisticolidae; Group III), 8 in *Sylvietta brachyura* (family Macrosphenidae; Group IV), 3 in *Panurus biarmicus* (family Panuridae; Group V), *Eremophila alpestris* (family Alaudidae; Group VI) and *Alauda arvensis* (family Alaudidae; Group VII), and, finally, 5 in *Alauda* (Group VII). The location of the NRRs for each group is displayed on the *Taeniopygia* reference chromosomes in Supplementary Fig. 1 and detailed in Supplementary Table 1. Notably, (i) the size of the NRRs of chromosome 4A and 3 varies between groups; (ii) parts of chromosome 5 is fused to chromosome Z in *Eremophila* (Group VI)^35^, but recombines over its entire length in the subspecies studied here (*E. a. flava*)^36^; and (iii) the recombination status of the ancestral PAR on chromosome Z within Sylvioidea remains unknown—a key focus of the present study.

### Recombination suppression of the ancestral PAR in three Sylvioidea lineages

We identified 23 ancestral PAR genes in the *Taeniopygia* reference genome and orthologs of these genes in the assemblies of all studied species (see Methods; Supplementary Table 2). Similar sequencing read coverage between sexes confirmed the presence of two gene copies for each of these 23 genes in both males and females (Supplementary Fig. 2). In contrast, genes located in the NRR adjacent to the PAR on chromosome Z, where females are hemizygous due to ancient W degeneration, showed substantially lower read coverage in females (Supplementary Fig. 2). To determine whether the ancestral PAR remains pseudoautosomal or has undergone recombination suppression in any species, we generated Z- and W-linked sequences for each ancestral PAR gene. For *Taeniopygia*, these sequences were extracted directly from Z and W reference scaffolds, which include the PAR. For the other species, we extracted Z- and W-linked PAR sequences based on single nucleotide polymorphism (SNP) detected in male and female short-read sequencing data (see Methods; Supplementary Table 3). In essence, recombination suppression is expected to lead to diverging Z and W sequences, resulting in an excess of unique SNPs in ZW females that represent unique W-linked variants. In contrast, when Z and W recombine, few sex-specific SNPs are expected. After alignment and filtering of short sequences (<500 bp), 17 of the 23 genes remained for analysis, including 13 protein-coding genes and four long non-coding RNAs (Supplementary Table 2). We then used the aligned Z and W sequences from all species to construct phylogenetic trees for each PAR gene and calculate intraspecific Z-to-W branch distances, defined as the sum of branch lengths separating the Z and W tips for each species (see Methods). Using this approach, recombination suppression across the ancestral PAR in a species can be inferred from elevated Z-to-W branch distances relative to those in *Taeniopygia*, in which the ancestral PAR is recombining. If recombination suppression predates species divergence, Z and W sequences are expected to cluster by chromosome rather than by species in the phylogeny (e.g., ref. ^51^).

**Fig. 1:**
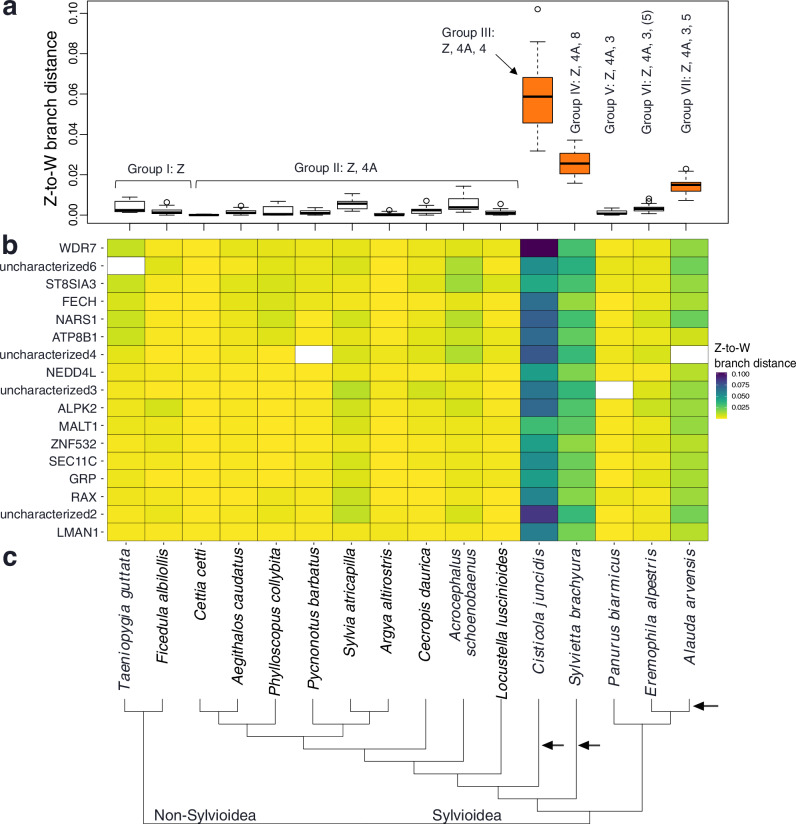
Recombination suppression in the ancestral PAR in three Sylvioidea species. **a** Boxplots showing Z-to-W branch distances for ancestral PAR genes (*n* = 17 genes) in each species (*n* = 16 species), based on phylogenetic tree branch lengths. Larger distances indicate greater divergence between Z and W copies, consistent with recombination suppression. Orange boxplots highlight species with significantly greater Z-to-W branch distances than *Taeniopygia* (see Supplementary Table 4). Above each boxplot, the combination of sex-linked chromosomes is indicated. Chromosomes shown in bold (chr. 4, 8, 5) are inferred to have fused to the PAR-end of chromosome Z. Boxplots display the median, interquartile range (25th–75th percentiles), whiskers (±1.5 × IQR) and outliers beyond the whiskers. **b** Z-to-W branch distance for individual ancestral PAR genes in each species. **c** Cladogram showing the phylogenic relationships among the studied species. Black arrows mark branches leading to species where ancestral PAR genes show evidence of recombination suppression.

In *Taeniopygia*, the PAR genes showed a median Z-to-W branch distance of 0.0024 (*n* = 16 genes; Fig. 1; Supplementary Table 4). Three Sylvioidea species exhibited significantly greater Z-to-W branch distances than *Taeniopygia* at these genes (two-sided Wilcoxon sign-rank tests with Bonferroni correction for multiple testing all 15 species; Fig. 1; Supplementary Table 4; Supplementary Data 1): *Cisticola* (median = 0.0587; *W* = 272, *n* = 17, *P* = 2.57 × 10^−8^), *Sylvietta* (median = 0.0255; *W* = 272, *n* = 17, *P* = 2.57 × 10^−8^) and *Alauda* (median = 0.0150; *W* = 252, *n* = 16, *P* = 5.99 × 10^−7^). These results suggest that recombination has ceased along the ancestral PAR in these species, i.e., the ancestral PAR has been lost. In contrast, *Ficedula* and the remaining 11 Sylvioidea species did not show significantly greater Z-to-W branch distances compared to *Taeniopygia* (Fig. 1; Supplementary Table 4; Supplementary Data 1). The Z and W sequences clustered within species, supporting that recombination suppression occurred independently in *Cisticola*, *Sylvietta* and *Alauda*. To further evaluate recombination cessation of the ancestral PAR, we compared the number of unique SNPs (i.e., genetic variants present only in one sample and in heterozygous form) between sexes for each gene within species (Supplementary Fig. 3). Females had significantly more unique SNPs than the males (two-sided paired Wilcoxon sign-rank tests with Bonferroni correction for testing 15 species; Supplementary Table 5) in *Cisticola* (female > males: all genes; paired *W* = 153, *n* = 17, *P* = 0.0048), *Sylvietta* (all genes; paired *W* = 153, *n* = 17, *P* = 0.0048), and *Alauda* (94% of genes; paired *W* = 136, *n* = 17, *P* = 0.0072). No significant sex difference in SNP counts was observed in any other species (*P* > 0.242; Supplementary Table 5). Together, these results provide strong support for independent loss of recombination of the ancestral PAR in three Sylvioidea lineages: *Cisticola* in family Cisticolidae (Group III), *Sylvietta* in family Macrosphenidae (Group IV), and *Alauda* in family Alaudidae (Group VII) (Fig. 1).

### Recombination suppression of the ancestral PAR is associated with, but is not directly caused by, fusions to the chromosome Z

Interestingly, the species with recombination-suppressed ancestral PAR each possess unique, non-recombining sex-linked chromosomes: chromosome 4 in *Cisticola*, chromosome 8 in *Sylvietta*, and chromosome 5 in *Alauda*. Fusions involving the PAR-end of chromosome Z, followed by the spread of recombination suppression into the translocated chromosome, could explain the observed loss of recombination in these species.

To investigate (i) which regions of the sex-linked chromosomes are fused to the sex chromosome or segregate autosomally, (ii) the structural organisation of the enlarged neo-sex chromosomes, and (iii) the presence of putative novel PARs in lineages where the ancestral PAR has become non-recombining, we analysed published high-quality reference genomes from selected Sylvioidea species. These included *Cisticola* (Group III), *Sylvietta virens* (Group IV; no high-quality reference genome was available for *S. brachyura*), *Eremophila* (Group VI) and *Alauda* (Group VII) (Supplementary Table 6). We searched for scaffolds informative of chromosomal fissions, fusions and novel PAR boundaries. Fusion scaffolds were defined as single scaffolds sharing synteny with two distinct chromosomes in the *Taeniopygia* reference genome. Fission scaffolds were defined as pairs of scaffolds flanking either side of an NRR-end, where one scaffold exhibits a sex-linked pattern (i.e., sex-specific sequencing read coverage and/or SNP count), and the other shows an autosomal pattern (i.e., no sex difference). PAR boundary scaffolds were defined as single scaffolds spanning an NRR-end, with one segment located within the NRR and the other in the PAR. Detailed synteny and sex-linkage information for all informative scaffolds is provided in Supplementary Figs. 4–9 and Supplementary Table 7.

**Fig. 2:**
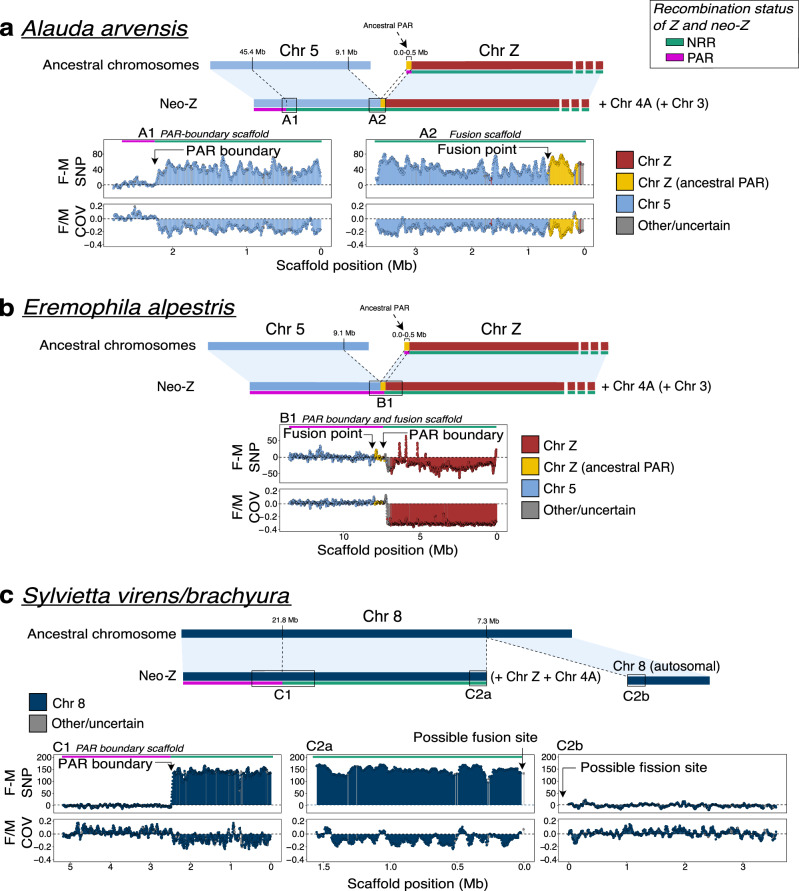
Representative examples of scaffolds supporting chromosomal fusions, fissions or PAR boundaries in Sylvioidea songbirds. **a** In *Alauda*, a PAR boundary scaffold (A1) shows complete collinearity with *Taeniopygia* chromosome 5 across the NRR-end at position 45.4 Mb (i.e., the location of the PAR boundary). A fusion scaffold (A2) shares synteny with both chromosome 5 and Z, with a contact point between the other chromosome 5 NRR-end (position 9.1 Mb) and the PAR-end of chromosome Z. **b** In *Eremophila*, a fusion scaffold (B1) spans the same fusion point between chromosome 5 and Z as in *Alauda*. However, in *Eremophila* (ssp. *E. a. flava*) neither the ancestral PAR nor chromosome 5 has ceased to recombine. This scaffold marks both the fusion point between chromosomes 5 and Z and the ancestral PAR boundary on chromosome Z. **c** In *Sylvietta virens*/*brachyura*, a PAR boundary scaffold (C1) spans the chromosome 8 NRR-end at position 21.8 Mb, while a pair of fission scaffolds (C2a, C2b) flank either side of the other NRR-end (position 7.3 Mb). **a**–**c** Each panel shows *Taeniopygia* reference chromosomes (ancestral chromosomes) and homologous regions in each Sylvioidea species, including chromosome names, ancestral or novel PARs (purple sections), Sylvioidea NRRs (green lines), scaffold locations (boxes), and additional sex-linked chromosomes in Sylvioidea (uncertain placements indicated in parentheses). Inserts display female–male SNP count difference (F-M SNP), and female-to-male read coverage ratio (F/M COV), in genomic windows across each scaffold, based on whole-genome short-read sequence data (see Methods). For detailed synteny information, see Supplementary Figs. 4–9.

For the three species with recombination-suppressed ancestral PAR (*Cisticola*, *Sylvietta* and *Alauda*), we found direct evidence supporting our hypothesis that the lineage-specific sex-linked chromosome fused with the PAR-end of chromosome Z in one case. Specifically, in *Alauda*, a fusion scaffold linked the ancestral PAR-end of chromosome Z (position 0.0 Mb) with an NRR-end on chromosome 5 (position 9.1 Mb) (Fig. 2a; Supplementary Fig. 4). This scaffold exhibited distinct sex-specific signature across its entire length, based on sequencing read coverage and SNP count (Fig. 2a), confirming recombination suppression of the ancestral PAR in the species. In all three species, we found evidence of a novel PAR on the lineage-specific sex-linked chromosome: PAR boundary scaffolds spanned an NRR-end on chromosome 5 (position 45.4 Mb) in *Alauda* (Fig. 2a; Supplementary Fig. 4), on chromosome 4 (position 49.1 Mb) in *Cisticola* (Supplementary Fig. 5), and on chromosome 8 (position 21.8 Mb) in *Sylvietta virens/brachyura* (Fig. 2c; Supplementary Fig. 6). Additionally, for both *Cisticola* and *Sylvietta*, we identified a pair of fission scaffolds flanking the opposite NRR-end: chromosome 4 (position 13.8 Mb) in *Cisticola* (Supplementary Fig. 5), and chromosome 8 (position 7.3 Mb) in *Sylvietta* (Fig. 2c; Supplementary Fig. 6). Given the location of the PAR boundary, we infer that these positions mark fusion points in *Cisticola* and *Sylvietta*, respectively.

In *Eremophila*, where no evidence of recombination suppression of the ancestral PAR was found (Fig. 1), we identified a fusion scaffold linking chromosome 5—at precisely the same position as in *Alauda* (9.1 Mb)—to the PAR-end of chromosome Z (Fig. 2b; Supplementary Fig. 7; see also ref. ^35^). Notably, the same scaffold is also a PAR boundary scaffold: it spans across chromosome 5 and the ancestral PAR, showing no sex-specific signal in sequencing read coverage and SNP count in these regions, and extends over the ancestral PAR boundary and into the NRR of chromosome Z, where sex-specific differentiation is evident. These findings suggest that the fusion between chromosome 5 and the PAR-end of chromosome Z is shared between *Eremophila* (Group VI) and *Alauda* (Group VII) in family Alaudidae. However, the evolutionary outcomes of this fusion differ between the two genera. In *Eremophila*, recombination was retained, resulting in an expanded PAR that includes parts of chromosome 5 and the ancestral PAR. In contrast, *Alauda* experienced recombination suppression, shifting the PAR boundary from its ancestral location on chromosome Z to a new position on chromosome 5 (45.4 Mb). This contrast supports the conclusion that recombination suppression of the ancestral PAR did not occur as a direct consequence of the chromosomal fusion, but rather through subsequent lineage-specific events.

Previous studies have shown that chromosome Z and a part of chromosome 4A fused early in the Sylvioidea lineage, specifically merging the non-PAR-end of chromosome Z (position 73.4 Mb) with an NRR-end of chromosome 4A (position 9.6 Mb)^31,32^. We confirmed this fusion in *Eremophila*, where a fusion scaffold spans these exact positions on Z and 4A (Supplementary Fig. 8; see also ref. ^35^). In *Sylvietta*, which exhibits a reduced NRR on chromosome 4A (5.3–9.6 Mb) compared to other Sylvioidea (0–9.6 Mb; Supplementary Fig. 1), we identified a novel PAR on chromosome 4A. This was supported by a PAR boundary scaffold spanning chromosome 4A position 5.3 Mb in *Sylvietta virens/brachyura* (Supplementary Fig. 9; see also ref. ^38^).

The complex pattern observed on chromosome 3, characterised by multiple NRRs of varying sizes in Groups V, VI and VII (Supplementary Fig. 1), suggests that chromosomal rearrangements have occurred between Sylvioidea and *Taeniopygia*. This is supported by synteny analysis, which revealed multiple rearrangements along chromosome 3 between *Eremophila* and *Taeniopygia* (Supplementary Fig. 8). Despite this complexity, we identified fusion scaffolds in both *Eremophila* and *Alauda* that link the NRR of chromosome 4A (position 0.0 Mb) to an NRR on chromosome 3 (position 24.1 Mb in both species; Supplementary Table 7). Furthermore, a PAR boundary scaffold in *Eremophila* confirmed a novel PAR boundary on chromosome 3 at position 14.0 Mb (Supplementary Fig. 8c, f; see also ref. ^35^). No PAR boundary scaffold was identified on chromosome 3 in *Alauda*, but the recombination-suppressed region in this species is substantially larger than in *Eremophila* (Supplementary Fig. 1), providing additional evidence for lineage-specific recombination dynamics.

**Fig. 3:**
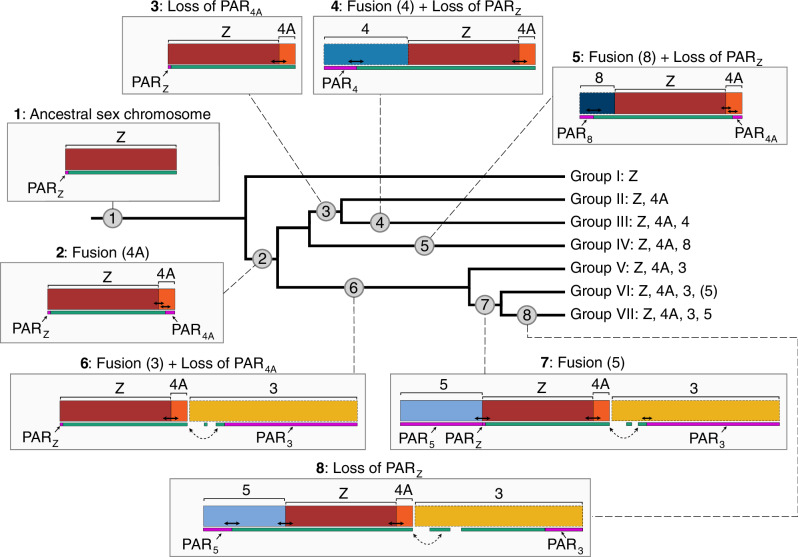
The enlarged neo-sex chromosomes in Sylvioidea. Phylogeny and schematic illustration of the hypothesised neo-Z chromosomal rearrangements in Sylvioidea. Chromosomes are numbered and shown in seperate colours, with NRRs indicated in green and PARs in pink. Horisontal arrows connecting chromosomes, or spanning across an NRR to a PAR, indicate scaffold-based evidence (dashed arrows connect chromosome 4A to the rearranged part of chromosome 3).

These findings indicate that the enlarged neo-sex chromosomes in Sylvioidea Groups II–VII consist of distinct segments of PAR(s) and NRRs, with their inferred organisation and boundaries summarised in Fig. 3 and detailed in Supplementary Table 8.

### Increasing Z-to-W PAR gene sequence divergence towards the ancestral PAR boundary

To test the prediction that Z-to-W sequence divergence increases with proximity to the ancestral PAR boundary^5,6,8^, we correlated the Z-to-W branch distances derived from the gene trees (Fig. 1; Supplementary Table 4) with the chromosomal positions of genes on the ancestral PAR (Supplementary Table 2; Supplementary Data 1). Gene positions and order were based on the *Taeniopygia* reference genome for all species. Gene order collinearity was confirmed in all species where the entire ancestral PAR is contained within a single scaffold (*Taeniopygia*, *Ficedula*, *Alauda* and *Sylvietta*; Supplementary Table 9; Supplementary Fig. 10). In contrast, two species with more fragmented assemblies (*Pycnonotus barbatus* and *Sylvia atricapilla*) showed non-collinearity at the beginning of the PAR, suggesting rearrangements from the ancestral gene order (Supplementary Fig. 11). For the remaining nine species, we found no evidence of non-collinearity with the *Taeniopygia* PAR region (Supplementary Fig. 11), although gene order certainty remains limited due to assembly fragmentation.

**Fig. 4:**
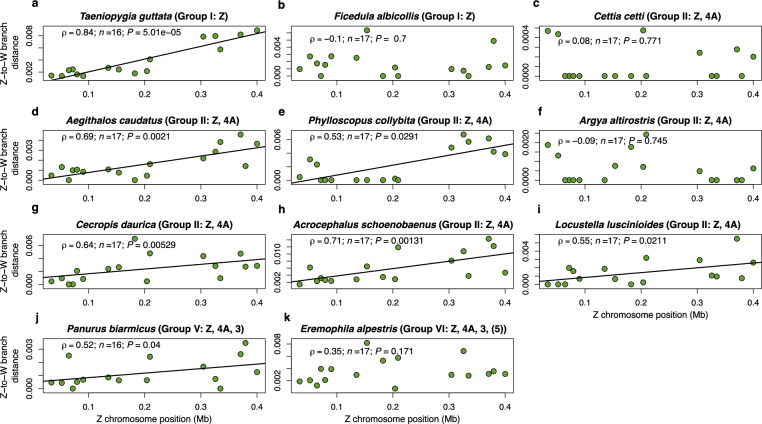
Z-to-W sequence divergence along the ancestral PAR. Relationship between Z-to-W branch distance and chromosomal position of ancestral PAR genes (*n* = 17 genes; 0 Mb = proximal end; 0.47 Mb = near the PAR boundary on the *Taeniopygia* Z chromosome) for (**a**, **b**) non-Sylvioidea species (Group I), **c**–**i** Sylvioidea species with a single sex chromosome–autosome fusion (Group II), and (**j**, **k**) Sylvioidea species with multiple fusions (Groups V–VI). Spearman’s rank correlation coefficient (*ρ*), number of genes (*n*), and two-sided *p* value (*P*) are reported for each species. Least square regression lines are shown for significant correlations. Note: No correlation is expected in species where the ancestral PAR is non-recombining or in species with non-collinear gene order relative to *Taeniopygia*; non-significant correlations for these species are shown in Supplementary Fig. 12.

In *Taeniopygia*, Z-to-W branch distances increased significantly with proximity to the PAR boundary (position 468 kb; two-sided Spearman’s rank correlation test: *ρ* = 0.838, *n* = 16 genes, *P* < 0.001; Fig. 4a; Supplementary Data 1). Among species with retained recombination and presumed gene collinearity with *Taeniopygia*, six showed significant correlations between Z-to-W branch distances and gene position (*ρ* = 0.517–0.713, *n* = 16–17 genes, *P* = 0.001–0.040). In contrast, *Ficedula* and four Sylvioidea species—including *Eremophila*, where a PAR-end fusion of chromosome 5 is confirmed—showed non-significant correlations (*ρ* = -0.101–0.348, *n* = 16–17 genes, *P* = 0.171–0.700) (Fig. 4b–k; Supplementary Table 10). Non-significant correlations were also observed in *Pycnonotus* and *Sylvia* (Supplementary Fig. 12a, b), which show signs of rearrangements relative the *Taeniopygia* gene order (Supplementary Fig. 11). Finally, the three species with recombination-suppressed ancestral PARs*—Cisticola*, *Sylvietta* and *Alauda*—also showed non-significant correlations (*ρ* = 0.167–0.333, *n* = 16–17 genes, *P* = 0.191–0.523; Supplementary Fig. 12c–e; Supplementary Table 10), consistent with region-wide W divergence due to recombination cessation.

## Discussion

Why some lineages retain largely homomorphic sex chromosomes with extended PARs (e.g., paleognaths^15,23,52^) while others exhibit near-complete recombination suppression across their sex chromosomes (e.g., songbirds^23,46–48^) remains an open question. Comparative studies that uncover the evolutionary dynamics of differentially structured PARs among closely related taxa may offer valuable insights into this disparity. In this study, we present a comprehensive analysis of the relationship between novel sex-linked regions and changes to the ancestral PAR in Sylvioidea passerine birds. This clade is characterised by enlarged neo-sex chromosomes formed through multiple sex chromosome–autosome fusions^29–39^, providing a unique framework to explore how recombination suppression and PAR restructuring evolve across lineages.

Our results reveal that the ancestral PAR, which is otherwise highly conserved among passerines^23,46–48^, has independently evolved recombination suppression in three distinct Sylvioidea lineages: *Cisticola*, *Sylvietta* and *Alauda*. Recombination suppression of the ancestral PAR in these three genera is supported by long Z-to-W branch distances, excess heterozygosity and elevated number of unique SNPs (27–125 SNPs) in ZW females. In contrast, species in which the ancestral PAR is not recombination suppressed show short Z-to-W branch distances, and similar level of heterozygosity and number of unique SNPs between sexes, with females having between −2 and 2 unique SNP compared to males. The magnitude and spatial consistency of these signals of recombination suppression far exceed what can plausibly be generated by random sequencing errors in short-read data. Notably, each instance of recombination suppression of the ancestral PAR is associated with distinct, lineage-specific sex-linked regions: parts of chromosome 4 in *Cisticola*, 8 in *Sylvietta*, and 5 in *Alauda*. A plausible scenario explaining these patterns is that the translocated chromosomes fused to the PAR-end of chromosome Z, facilitating the spread of recombination suppression from the ancestral PAR into the newly fused chromosome. This hypothesis is support by direct evidence of physical linkage between chromosome 5 and the PAR-end of chromosome Z in *Alauda* (Figs. 2a, 3), where the PAR boundary has shifted from chromosome Z to a new location on chromosome 5. Although available scaffold data do not allow precise positioning of chromosomes 4 and 8 in *Cisticola* and *Sylvietta*, respectively, we hypothesise that these chromosomes also fused to the PAR-end of the Z chromosome, after which recombination suppression spread and formed new PARs in the fused chromosomes (Fig. 3). Data from long-read sequencing, karyotyping or pedigree-based linkage mapping would be required to confirm these fusion points, but such data are challenging to obtain because they would require extensive fieldwork to collect suitable material. However, in *Sylvietta*, this chromosome arrangement is supported by the presence of a second PAR at the opposite end of the sex chromosome, within the translocated segment of chromosome 4A (Fig. 3; see also ref. ^38^). Interestingly, as in *Alauda*, scaffold data also indicate physical linkage between chromosomes 5 and Z in another lark genus, *Eremophila* (Figs. 2b, 3; see also ref. ^35^), where neither the ancestral PAR nor parts of chromosome 5 have ceased to recombine (Figs. 1, 2b). This contrast indicates that recombination suppression of the ancestral PAR was not a direct consequence of the PAR-end fusion itself, but instead evolved through subsequent, lineage-specific processes. The situation in *Eremophila* is further complicated by subspecies variation: two North American subspecies (*E. a. lamprochroma* and *E. a. ammophila*) show recombination suppression across a small region of chromosome 5^35^, unlike the Palearctic subspecies (*E. a. flava*) studied here. These subspecies diverged approximately 0.7 MYA^53^, suggesting ongoing recombination dynamics within this lineage.

We confirmed the fusion point between the non-PAR end of chromosome Z and the distal NRR-end of chromosome 4A, consistent with previous findings in Sylvioidea^31,32^. Additionally, we found support for a fusion between the proximal NRR-end of chromosome 4A and the NRR of chromosome 3 in *Panurus*, *Eremophila* and *Alauda* (groups V–VII; Fig. 3). As Panuridae (*Panurus*) and Alaudidae (*Eremophula* and *Alauda*) are sister families, this suggests a single fusion event between chromosomes 4A and 3.

Based on all confirmed and suggested fusions and NRRs across Sylvioidea (Fig. 3), we hypothesise that novel and sometimes extensive PARs have formed and been retained on translocated chromosomes in several species. These include chromosome 4 in *Cisticola* (Group III), 4A and 8 in *Sylvietta* (Group IV), 3 in *Panurus*, *Eremophila* and *Alauda* (Group V–VII), and 5 in *Eremophila* and *Alauda* (Group VI–VII). If the two large PARs on chromosomes 3 and 5 proposed for *Eremophila* are correct, they would represent the largest PAR described in birds to date (>110 Mb), more than twice the size of the PAR of *Struthio camelus* (52 Mb)^54^. This hypothesis is supported by the unusually large Z chromosome in the *Eremophila* karyotype^55^. Future studies using more contiguous reference genomes are needed to confirm the exact fusion points and extent of these proposed PARs in Sylvioidea. Furthermore, assembling the W chromosome and verifying linkage of chromosomes 3, 4, 5 and 8 to the ancestral W chromosome, as has been shown for chromosome 4A^31,32^, could provide deeper insights into the mechanisms driving recombination suppression and PAR transitions on these sex chromosomes.

Evidence from a linkage map in another Sylvioidea species, *Acrocephalus arundinaceus*, shows that the distal part of chromosome 4A (9.6–20.7 Mb), which does not show signs of recombination suppression, segregates as a separate autosome^56^. This region also contains the centromere, according to data from *Taeniopygia*^57^ (Supplementary Fig. 1). For the translocated chromosomes 4, 8 and 5 in *Cisticola*, *Sylvietta* and *Alauda* (and *Eremophila*), respectively, we propose that the recombining regions flanking the putative fission breakpoints are likewise segregating as separate autosomes in each lineage. These presumed autosomal regions also contain the suggested centromere in *Taeniopygia*^57^, supporting their independent segregation. The situation is more complex for chromosome 3, which shows intrachromosomal rearrangements and varying degree of recombination suppression in *Panurus*, *Eremophila* and *Alauda*. This complexity makes it difficult to infer which part of the chromosome, if any, segregates autosomally in these lineages. Notably, the centromere position in *Taeniopygia*^57^ lies within the NRR of *Alauda*, suggesting that the centromere may have shifted in species where parts of chromosome 3 have fused to the sex chromosomes.

Theory predicts that expansions of recombination suppression along sex chromosomes are preceded by increasing sexually antagonistic sequence divergence in regions of the PAR near the PAR boundary^5,8^. However, sex-associated neutral genetic variation is also expected to accumulate close to the PAR boundary because of cosegregation with Z and W^5,6,8^. A key factor influencing the extent of sex-associated polymorphism within PAR is the recombination rate^5,8,16,21^. In passerines, the ancestral PAR is characterised by high recombination rate, owing to its small size and obligate crossover in females^47,56^. This configuration is expected to restrict Z-to-W divergence to a narrow region near the PAR boundary. Consistent with this prediction, we detected statistically significant correlations between Z-to-W branch distances and proximity to the PAR boundary in seven of the eleven species where the ancestral PAR is retained and where conserved PAR gene order was either confirmed or not rejected (Fig. 4). In contrast, no such correlation was observed in species when not expected—namely, in the three species exhibiting recombination suppression in this region (*Cisticola*, *Sylvietta* and *Alauda*) and in two species for which conserved gene order was refuted (*Pycnonotus* and *Sylvia*; Supplementary Fig. 12). The absence of correlation between Z-to-W branch distances and proximity to the PAR boundary in four of the eleven species where it could be expected (Fig. 4) is not surprising and may be explained by several factors. First, we used the PAR gene order from the *Taeniopygia* reference genome^49,50^ for all species, although incomplete scaffolding of the ancestral PAR in several species means that their true gene order remains unknown. The *Taeniopygia* gene order was confirmed in three species (*Ficedula, Alauda* and *Sylvietta*), but refuted in two (*Pycnonotus* and *Sylvia*). Unaccounted rearrangements may therefore explain the weak or absent correlation in some species (Fig. 4), such as in *Pycnonotus* and *Sylvia* (Supplementary Fig. 12). Future work will be required to establish the PAR gene order in species where it is currently unresolved, ideally using long-read sequencing data, which may help explain the lack of correlation in some cases. Second, the theoretical framework for selection-driven recombination suppression assumes the presence of sexually antagonistic genetic variation^8^, which remains to be empirically demonstrated in these species. The long-term retention of the ancestral PAR in passerines may instead suggest that the observed increase in sex-associated divergence near the PAR boundary primarily reflects neutral processes rather than sexually antagonistic variation. If sexually antagonistic selection were widespread, we might expect progressive shrinkage of the ancestral PAR in at least some species. Alternatively, constraints on the minimum size of the PAR, such as requirements for proper segregation of the sex chromosomes, may prevent PAR reduction even in the presence of sexually antagonistic selection. PAR-end fusions could have released the ancestral PAR from such constraints, allowing the PAR boundary to shift into the newly sex-linked regions. Such shifts may have been facilitated by the fusion drastically lowering the recombination rate and capturing sexually antagonistic polymorphism either in the ancestral PAR or in the large, gene-rich translocated region^58,59^. Notably, we detected PAR-boundary movement in three of the four species with inferred PAR-end fusions (*Cisticola*, *Sylvietta* and *Alauda*). In contrast, the species with PAR-end fusion but maintained ancestral PAR, *Eremophila*, did not show increasing Z-to-W divergence near the PAR boundary within the ancestral PAR region (Fig. 4k).

In conclusion, our study reveals exceptional levels of sex chromosome–autosome translocation and PAR diversity in Sylvioidea songbirds, offering important insights into the evolutionary dynamics of recombination suppression. We show that the otherwise highly conserved ancestral PAR^23,46–48^ has ceased to recombine independently in three genera: *Cisticola*, *Sylvietta* and *Alauda*. Each of these cases was associated formation of novel PARs in uniquely translocated regions. Notably, *Eremophila* shares the fusion of chromosome 5 to the ancestral PAR-end with its sister genus *Alauda*, yet retains the ancestral PAR (at least in the subspecies *E. a. flava* studied here; the situation might differ for North American subspecies^35^). This contrast suggests that the fusion itself did not directly cause recombination suppression across the ancestral PAR, but rather enabled it through lineage-specific processes. Our data further suggest that *Sylvietta*, *Alauda* and *Eremophila* possess two PARs, a feature not know to occur among other birds. Finally, we provide empirical support for the hypothesis that episodes of recombination suppression are preceded by increasing Z-to-W sequence divergence near the PAR boundary. We propose that PAR-end fusions have released the ancestral PAR from constraints in evolving recombination suppression, enabling the PAR boundary to shift into the newly sex-linked region.

## Methods

### Identification of PAR genes

The ancestral songbird PAR was first characterised in *Ficedula*, where identified as a ~ 700 kb genomic region at the beginning of the Z chromosome, spanning three scaffolds^46^. This region shares synteny with the proximal 468 kb of the scaffold Z_random in the *Taeniopygia* reference genome v. 3.2.4 (Taeniopygia_guttata-3.2.4; NCBI RefSeq assembly GCA_000151805.2)^49^, which has also been shown to be pseudoautosomal^41^. In a recent genome assembly of *Taeniopygia* (bTaeGut2.pat.W.v2; NCBI RefSeq GCF_008822105.2)^50^, the PAR is integrated as the first 468 kb of the Z chromosome (NC_045027.1), and the W-linked PAR copy is included as a separate scaffold (NW_022611471.1). To maintain consistent chromosomal positioning of NRRs with previous studies in Sylvioidea^32,36–39^, we performed chromosome-level analyses using v. 3.2.4 (ref. ^49^), which we had modified by placing parts of the previously unanchored scaffold containing the PAR (Z_random:0–468 kb) at the beginning of chromosome Z (an adjustment aligning with bTaeGut2.pat.W.v2; ref. ^50^).

To define the PAR gene set, we first extracted the longest transcript for each gene in the *Taeniopygia* annotation (genome version bTaeGut2.pat.W.v2) using the *CGAT* (v0.3.3) tool *gtf2gtf* (option --method=filter --filter-method=longest-transcript)^60^. After filtering, we identified transcripts from 23 genes (15 protein-coding genes, 7 long non-coding genes, and 1 microRNA) located within the first 468 kb of the Z chromosome (NC_045027.1:0–468 kb), corresponding to the PAR (Supplementary Table 2). We then annotated these genes in available assemblies of the 14 Sylvioidea species^32,36–39^ (previously assembled by us from the paired-end short-read sequencing data of males listed in Supplementary Table 3) and in the reference genome of *Ficedula* (FicAlb1.5; NCBI RefSeq assembly: GCA_000247815.2) by employing an annotation lift-over approach. This involved synteny analyses between each assembly and the *Taeniopygia* Z chromosome using *SatsumaSynteny* v2.0^61^. Gene annotations from *Taeniopygia* were transferred to the Sylvioidea and *Ficedula* assemblies using *kraken*^62^, enabling identification of exon coordinates for each PAR gene in all species.

To identify the W-copies of the PAR genes in the *Taeniopygia* genome, we first constructed gene sequences from the longest transcript of each gene using *gffread* (DOI: 10.5281/zenodo.3739573) (option -w). Next, gene sequences located on the W-linked PAR scaffold (NW_022611471.1) were blasted against all gene sequences using *blastn*+ 2.9.0^63^, and those that aligned to Z-linked PAR genes with an E-value of 0.0 were classified as the W-copy of that gene. This approach resulted in 18 W-linked genes in *Taeniopygia*, comprising 15 protein-coding genes and 3 long non-coding genes.

### Extracting Z and W PAR gene sequences in Sylvioidea and Ficedula

We extracted Z and W sequences of ancestral PAR genes from paired-end short-read sequencing data of male and female individuals for each of the other 15 species (14 Sylvioidea species and *Ficedula*; Supplementary Table 3). The sequencing reads of the individuals were aligned to the assemblies of respective species (previously assembled; refs. ^32,36–39^) using *bwa mem* v0.7.17^64^, *samtools* v1.7^65^ and *picardtools* v2.18.0 (http://broadinstitute.github.io/picard/). Variant calling was performed using *freebayes* v1.1.0^66^, and the resulting VCF file was filtered with *vcftools* v0.1.14^67^ (setting --non-ref-ac 1 --min-alleles 2 --max-alleles 2 --remove-filtered-all --minQ 20 --minDP 5). Consensus sequences for each sex were extracted for each PAR gene and exon using *bcftools consensus* (v1.9), replacing reference base pairs with the alternative allele. To reverse complement exons annotated on the minus (-) strand in the lift-over analysis, we applied *fastx_reverse_complement* from the *FASTX* toolkit (v0.0.14; http://hannonlab.cshl.edu/fastx_toolkit). These sex-specific consensus sequences were used to represent Z and W sequences.

To confirm the presence of two gene copies in both females and males at PAR genes, we compared female-to-male sequencing read coverage ratios for all gene across the Z chromosome. For each individual, we first calculated per-exon read coverage from aligned paired-end reads (Supplementary Table 3) using *bamstat04* from the *jvarkit* toolbox^68^, and then the mean coverage per gene using exons with coverage between 5 and 80. Next, female-to-male read coverage ratios were calculated per gene for each species, removing genes with unexpectedly high ratios (cutoff 2.5). Within each species, each gene-specific ratio was then (i) normalised by dividing with the mean ratio of all genes across the Z chromosome, and (ii) standardised by subtracting this mean. Because almost all Z-linked genes lack a W homologue, the chromosome-wide mean approximates the value for genes with a single copy in females. Under this normalisation and standardisation, genes with one copy in females (as expected for older sex chromosome regions where W is lost) are expected to have values near zero, whereas genes with two copies in females (as expected for PAR genes with both Z and W copies) should have values close to one – consistent with our observation (Supplementary Fig. 2).

To compare the number of unique SNPs (variants present in only in one sample and appearing in heterozygous form) between sexes within species, we identified such variants in each gene using *vcftools* v.0.1.16^67^ (option –singletons).

### PAR gene tree analyses and Z-to-W branch distances

Z and W sequences of all species (excluding *Taeniopygia*) were aligned per exon using *prank* v.170427^69^ (option -F). The exon alignments were concatenated in the correct order for each PAR gene using the script *catfasta2phyml.pl* (https://github.com/nylander/catfasta2phyml.git). The Z and W sequences of *Taeniopygia* were then added to the alignments using *mafft* (v7.407; option --reorder --add --auto). The alignments were trimmed using *trimAl* v1.4.rev15^70^ (option -automated1). In two genes, extremely short (<200 bp) or poorly aligned sequences were removed from the alignments (*Panurus* in uncharacterized3, and *Pycnonotus* in uncharacterized4). Further trimming was performed using *trimAl* (setting -gt 0.8), removing sites with gaps in more than 20% of the sequences. Genes shorter than 500 bp were excluded, resulting in a final set of 16–17 genes per species (Supplementary Tables 2 and 4).

Maximum likelihood phylogenetic gene trees were constructed using *iqtree* v2.3.5^71^ (setting -m TEST -bb 1000 -alrt 1000). This setting invokes *ModelFinder*^72^ for automatic model selection, followed by tree constructing with ultrafast bootstrap support^73^. We calculated branch distances in the resulting gene trees using the *cophenetic.phylo* function in the R package *ape* v5.3^74^. We used the Z-to-W branch distances for each species, defined as the sum of branch lengths separating the Z and W tips, in the analyses.

### Identification of fusion, fission and PAR boundary scaffolds

We previously analysed genomic data from the 14 Sylvioidea species to identify sex-linked genomic regions using a combination of sex-specific sequencing read coverage and SNP counts^32,36–39^ (Supplementary Fig. 1). Genomic regions with a markedly reduced female-to-male read coverage ratio (F/M COV), and little or no female–male SNP count difference (F-M SNP), are characteristic of sex-linked regions with high W chromosome degeneration (older sex chromosomes)^75,76^. Conversely, regions with similar F/M COV-values, but high F-M SNP-values, indicate sex-linked regions with limited W degeneration (younger sex chromosomes)^75,76^. Using these criteria, we previously identified partial or complete sex-linkage in six different chromosomes across the Sylvioidea clade, resulting from multiple sex chromosome–autosome fusions (chromosomes Z, 3, 4, 4A, 5 and 8; for details, see ref. ^37^).

One of the aims of this study was to identify fission breakpoints, fusion points and novel PAR boundaries associated with these translocations. However, earlier short-read based assemblies^32,36–39^ were too fragmented to support such analyses. To overcome this limitation, we obtained more contiguous published reference genomes for *Alauda*, *Cisticola*, *Eremophila* and *Sylvietta* (accession numbers in Supplementary Table 6). Because the fusion point between chromosomes Z and 4A is already known (see above), we focussed on Sylvioidea species with additional documented fusions. These reference genomes replaced our earlier fragmented assemblies^32,36–39^ for re-assessing sex linkage using the same approach (analysis of F/M COV and F-M SNP). Specifically, for each 5 kb genome window, we calculated sex-specific read coverage values from aligned BAM files using *bedtools* v2.27.1^77^. Female-to-male read coverage ratios (F/M COV) were calculated per window and then standardised by subtracting the genome-wide mean from each window-specific value. Under this standardisation, windows with equal coverage in the sexes should have values close to zero (representing autosomes and PAR), whereas windows with halved coverage in females should have values close to −0.5 (representing older sex chromosome regions where the W copy is lost). For the same windows, we quantified the number of unique SNPs for each sex in species-specific VCF files using *vcftools*^67^ (option –singletons). For each window, we subtracted the male SNP counts from the female SNP counts (F-M SNP). To improve visualisation, we applied a rolling mean across 10 windows for both F/M COV and F-M SNP values in Fig. 2, while Supplementary Figs. 4–9 show window-specific values. For *Sylvietta virens*, where paired-end data were unavailable, we used data from *Sylvietta brachyura*, its closest relative in our dataset.

To establish synteny between the reference genomes (Supplementary Table 6) and the chromosome-level genome assembly of *Taeniopygia* (v. 3.2.4), we used *lastal* from *last* v876^78^.

### Statistics and reproducibility

Details about samples (sample name and NCBI accession number) used in the analyses performed in this study are given in Supplementary Table 3. The study is based on 14 Sylvioidea species and two outgroup species (*Ficedula* and *Taeniopygia*). For each of these species, we use whole-genome short-read sequence data of one individual per sex (Supplementary Table 3; refs. ^36–39,46^) with the exception of *Taeniopygia* for which the sequencing data was directly extracted from the Z and the W chromosome (see Methods). Details about analyses and software are provided in Methods and at https://github.com/hsigeman/PAR. The statistical significance of difference in (i) Z-to-W branch distances and (ii) number of unique SNPs between each species (*n* = 15 species) and *Taeniopygia* was determined using two-sided Wilcoxon sign-rank tests with Bonferroni correction for multiple testing. The statistical significance of the correlation between Z-to-W branch distances and gene position was determined using two-sided Spearman’s rank correlation test for each species separately.

### Reporting summary

Further information on research design is available in the Nature Portfolio Reporting Summary linked to this article.

## Supplementary information

Supplementary Information

Description of Additional Supplementary Files

Supplementary Data 1

Reporting Summary

## Data Availability

All sequence data used in this study are publicly available on NCBI (short-read data: BioProjects PRJNA579268 and PRJEB7359; genome assemblies: accession numbers GCA_902810485.1, GCA_013400215.1, GCA_009792885.1, GCA_013399515.1, GCA_000151805.2, GCF_008822105.2, GCA_000247815.2). Sample information and NCBI accession numbers are provided in Supplementary Tables 3 and 6 (see also refs. ^36–39,46^). Source data underlying Figs. 1 and 4 are provided in Supplementary Data 1.

## Code availability

We have only used publicly available software (see Methods and GitHub (https://github.com/hsigeman/PAR)). Custom code and scripts used to handle sequence data, extract genomic data, launch software, produce figures and perform statistical analyses are provided at GitHub (https://github.com/hsigeman/PAR) and archived in Zenodo^79^.
