# Supplementary material for: Evolutionary dynamics of enlarged neo-sex chromosomes and novel pseudoautosomal regions in Sylvioidea songbirds
Source: Commun Biol. 2026 Jul 4;9:898. doi: 10.1038/s42003-026-10603-3 (PMC13333021; doi:10.1038/s42003-026-10603-3)
Supplement: Supplementary file 1 — Supplementary Information [file 42003_2026_10603_MOESM1_ESM.docx]

## **Supplementary Material**

## Evolutionary dynamics of enlarged neo-sex chromosomes and novel pseudoautosomal regions in Sylvioidea songbirds

Hanna Sigeman^a,b,c^, Simon J. Ellerstrand^a^, Bengt Hansson^a,#^

^a^ Department of Biology, Science for Life Laboratory, BECC – Biodiversity and Ecosystem services in a Changing Climate, Lund University, Lund, Sweden

^b^ Ecology and Genetics Research Unit, University of Oulu, Oulu, Finland

^c^ Department of Medical Biochemistry and Microbiology, Uppsala University, Uppsala, Sweden

# Corresponding author: Bengt Hansson ([bengt.hansson@biol.lu.se](mailto:bengt.hansson@biol.lu.se))

**Table of Contents**

Pages 2–13: Supplementary Figures 1–12

Pages 14–27 Supplementary Tables 1–10

Page 28 Supplementary References

**Supplementary Figure 1**: Sex-linked chromosomes and non-recombining regions (NRRs) in Sylvioidea songbirds. (**a**) Phylogenetic tree of the studied species, grouped into seven categories (I-VII) based on shared NNRs, as established in previous studies. Next to each group number are the names of the sex-linked chromosome, following the *Taeniopygia guttata* genome structure. In addition to chromosome Z shared by both non-Sylvioidea and Sylvioidea species (Group I-VII), chromosomes 4A is sex-linked only in Sylvioidea (Groups II-VII). Additional sex-linked chromosomes are restricted to specific Sylvioidea lineages: chromosome 4 (Group III), 8 (Group IV), 3 (Groups V-VII) and 5 (Group VI-VII). For *Eremophila alpestris*, chromosome 5 is shown in parenthesis, as it is fused to chromosome Z but does not exhibit NRRs in the subspecies studied here (*E. a. flava*). (**b**) Distribution of NRRs across chromosomes in each species group (see Supplementary Table 1 for details). The ancestral songbird PAR is located at the proximal end of the Z chromosome. Black diamonds within chromosomes indicate the approximate position of the centromere, as identified in the *Taeniopygia* reference genome.

**Supplementary Figure 2**: Female-to-male sequencing read coverage ratio at genes across the proximal 10 Mb of chromosome Z (n = 113-132 genes). The ancestral pseudoautosomal region (PAR; 0–0.5 Mb; n = 15-17 genes) is shaded in grey. Coverage ratios were normalised and standardised so that genes with one copy in females and two in males are expected to have values near zero, whereas genes with two copies in both females and males should have values close to one (see Methods). PAR genes exhibit similar read coverage in males and females, whereas genes in the non-recombining region (NRR; 0.5–10 Mb) show reduced coverage in females. This pattern suggests that PAR genes retain copies on both Z and W, and confirms that the W-linked copy has been lost for most genes in the NRR.

**Supplementary Figure 3**: Number of unique SNPs (variants present only in one sex and in heterozygous form) at PAR genes (n = 17 genes) in the female and the male in each species (n = 15 species). Females show significantly more unique SNPs than males – indicative of non-recombining PAR – in *Alauda arvensis*, *Sylvietta brachyura* and *Cisticola juncidis* (Supplementary Table 5). Note that gene lengths vary (Supplementary Table 2), which partly influences the number of SNPs occurring.

**Supplementary Figure 4**: Synteny between *Alauda arvensis* and *Taeniopygia guttata*. **(a,b)** Synteny between *Alauda* scaffolds (“CADDxxx”) and *Taeniopygia*, showing (a) chromosome 5 and (b) scaffold Z random, which contains the PAR within the first ∼0.5 Mb. The *Alauda* scaffolds are shown in the orientation given in the assembly (therefore some scaffolds are “inverted” relative to the *Taeniopygia* reference). Arrows mark scaffolds informative of the (c,e) fusion point and the (d) novel PAR boundary, respectively. (**c–e**) Female–male SNP count difference (F–M SNP) and female-to-male read coverage ratio (F/M COV), calculated in genomic windows (5 kb) across scaffold (c,e) CADDXX010000025.1 (syntenic to both chromosome Z and chromosome 5; fusion point) and (d) CADDXX010000058.1 (syntenic to chromosome 5; novel PAR boundary). Red data points in (a–d) exceed the genome-wide 95% confidence interval of F–M SNP or F/M COV. In (e), data points are coloured according to synteny with *Taeniopygia* chromosomes (scaffold Z random in red; chromosome 5 in blue). Note: F/M COV is standardised so that windows with equal coverage in the sexes have values close to zero (representing autosomes and the PAR), whereas windows with half the coverage in females have values close to −0.5 (representing older sex chromosome regions where the W copy is lost; see Methods).

**Supplementary Figure 5**: Synteny between *Cisticola juncidis* and *Taeniopygia guttata*. (**a**) Synteny between *Cisticola* scaffolds (“scaffoldxxx”) and *Taeniopygia* chromosome 4. The *Cisticola* scaffolds are shown in the orientation given in the assembly (therefore some scaffolds are “inverted” relative to the *Taeniopygia* reference). Arrows mark scaffolds informative of the (b) novel PAR boundary and the (c,d) fission point, respectively. (**b–d**) Female–male SNP count difference (F–M SNP) and female-to-male read coverage ratio (F/M COV), calculated in genomic windows (5 kb) across *Cisticola* scaffolds for (b) scaffold195 (novel PAR boundary), (**c**) scaffold687 (fission point; sex-linked part) and (d) scaffold2399 (fission point; autosomal part). Red data points in (a–d) exceed the genome-wide 95% confidence interval of F–M SNP or F/M COV. Note: F/M COV is standardised so that windows with equal coverage in the sexes have values close to zero (representing autosomes and the PAR), whereas windows with half the coverage in females have values close to -0.5 (representing older sex chromosome regions where the W copy is lost; see Methods).

**Supplementary Figure 6**: Synteny between *Sylvietta virens* and *Taeniopygia guttata* chromosome 8. (**a**) Synteny between *Sylvietta* scaffolds (“scaffold xxx”) and *Taeniopygia* chromosome 8. The *Sylvietta* scaffolds are shown in the orientation given in the assembly (therefore some scaffolds are “inverted” relative to the *Taeniopygia* reference). Arrows mark scaffolds informative of the (b) novel PAR boundary and the (c,d) fusion point, respectively. (**b–d**) Female–male SNP count difference (F–M SNP) and female-to-male read coverage ratio (F/M COV), calculated in genomic windows (5 kb) across *Sylvietta* scaffolds for (b) scaffold 32 (novel PAR boundary), (c) scaffold 170 (fission point; sex-linked part) and (d) scaffold 60 (fission point; autosomal part). Red data points in (a–d) exceed the genome-wide 95% confidence interval of F–M SNP or F/M COV. Note: F/M COV is standardised so that windows with equal coverage in the sexes have values close to zero (representing autosomes and the PAR), whereas windows with half the coverage in females have values close to -0.5 (representing older sex chromosome regions where the W copy is lost; see Methods).

**Supplementary Figure 7**: Synteny between *Eremophila alpestris* and *Taeniopygia guttata* chromosomes 5 and Z. (**a–c**) Synteny between *Eremophila* scaffolds (“WMCFxxx”) and *Taeniopygia* showing (a) chromosome 5, (b) scaffold Z random, which contains the PAR within the first ∼0.5 Mb, and (c) the remainder of chromosome Z. The *Eremophila* scaffolds are shown in the orientation given in the assembly (therefore some scaffolds are “inverted” relative to the *Taeniopygia* reference). Arrows mark the scaffold informative of the (d,e) fusion point between chromosomes 5 and Z. (**d,e**) Female–male SNP count difference (F–M SNP) and female-to-male read coverage ratio (F/M COV), calculated in genomic windows (5 kb) across *Eremophila* scaffold WMCF01000023.1 (syntenic to both chromosome Z and chromosome 5; fusion point). Red data points in (a–d) exceed the genome-wide 95% confidence interval of F–M SNP or F/M COV. In (e), data points are coloured according to synteny with *Taeniopygia* chromosomes (chromosome Z in green; scaffold Z random in red; chromosome 5 in blue). Note: F/M COV is standardised so that windows with equal coverage in the sexes have values close to zero (representing autosomes and the PAR), whereas windows with half the coverage in females have values close to -0.5 (representing older sex chromosome regions where the W copy is lost; see Methods).

**Supplementary Figure 8**: Synteny between *Eremophila alpestris* and *Taeniopygia guttata* chromosomes Z and 4A. (**a–c**) Synteny between *Eremophila* scaffolds (“WMCFxxx”) and *Taeniopygia* showing (a) chromosome Z, (b) chromosome 4A and (c) chromosome 3. The *Eremophila* scaffolds are shown in the orientation given in the assembly (therefore some scaffolds are “inverted” relative to the *Taeniopygia* reference). Arrows mark scaffolds informative of the (d,e) fusion points and the (f) novel PAR boundary, respectively. (**d–f**) Female–male SNP count difference (F–M SNP) and female-to-male read coverage ratio (F/M COV), calculated in genomic windows (5 kb) across *Eremophila* scaffolds for (d,e) WMCF01000024.1 (syntenic to chromosomes Z, 4A and 3; fusion points) and (f) WMCF01000011.1 (containing the novel PAR boundary on chromosome 3). Red data points in (a–d,f) exceed the genome-wide 95% confidence interval of F–M SNP or F/M COV. In (e), data points are coloured according to synteny with *Taeniopygia* chromosomes (chromosome Z in green; chromosome 4A in red; chromosome 3 in blue). Note: F/M COV is standardised so that windows with equal coverage in the sexes have values close to zero (representing autosomes and the PAR), whereas windows with half the coverage in females have values close to -0.5 (representing older sex chromosome regions where the W copy is lost; see Methods).

**Supplementary Figure 9**: Synteny between *Sylvietta virens* and *Taeniopygia guttata* chromosome 4A. (**a**) Synteny between *Sylvietta* scaffolds (“scaffold xxx”) and *Taeniopygia* chromosome 4A. The *Sylvietta* scaffolds are shown in the orientation given in the assembly (therefore some scaffolds are “inverted” relative to the *Taeniopygia* reference). The arrow marks a scaffold informative of the (b) novel PAR boundary. (**b**) Female–male SNP count difference (F–M SNP) and female-to-male read coverage ratio (F/M COV), calculated in genomic windows (5 kb) across *Sylvietta* scaffold 297 (containing the novel PAR boundary). Red data points in (a,b) exceed the genome-wide 95% confidence interval of F–M SNP or F/M COV. Note: F/M COV is standardised so that windows with equal coverage in the sexes have values close to zero (representing autosomes and the PAR), whereas windows with half the coverage in females have values close to -0.5 (representing older sex chromosome regions where the W copy is lost; see Methods).

**Supplementary Figure 10**: Synteny between PAR scaffolds of *Alauda arvensis*, *Sylvietta virens*, *Taeniopygia guttata* (W and Z PAR of assembly v. bTaeGut2.pat.W.v2; Z PAR of assembly v. 3.2.4) and *Ficedula albicollis* (accession numbers: Supplementary Table 6). Plot produced with AliTV [1].

**Supplementary Figure 11**: Synteny between scaffolds in the 14 Sylvioidea reference genomes (assembled from the male samples in Supplementary Table 3) and *Taeniopygia guttata* PAR. Scaffold boundaries are marked with dashed lines. Blue and grey data points mark scaffolds containing and not containing PAR genes, respectively. The scaffolds are shown in the orientation given in the assembly (therefore, some scaffolds are “inverted” in relation to the *Taeniopygia* reference). Red arrows mark scaffolds showing evidence of non-collinearity, suggesting rearrangements from the ancestral gene order, in *Pycnonotus barbatus* and *Sylvia atricapilla*.

**Supplementary Figure 12**: Relationship between Z-to-W branch distance and chromosomal position of ancestral PAR genes (n = 17 genes; 0 Mb = proximal end; 0.47 Mb = near the PAR boundary on the *Taeniopygia guttata* Z chromosome) for Sylvioidea species (**a,b**) with established non-collinearity to the gene order in *Taeniopygia* (*Pycnonotus barbatus* and *Sylvia atricapilla*) or (**c–e**) where the ancestral PAR is no longer recombining (*Cisticola*, *Sylvietta* and *Alauda*). Spearman’s rank correlation coefficient (ρ), number of genes (*n*), and two-sided p-value (*P*) are reported for each species. See Figure 4 for all other species.

| **Supplementary Table 1.** Ancestral chromosomal positions, according to the *Taeniopygia guttata* genome structure, of non-recombining regions (NRRs) detected in previous studies across the Sylvioidea clade. Group numbers correspond to those in Figure 1. The positions of the two outermost edges for each coherent NRR (“NRR edges”) are given as the proximal edge being the one closer to the beginning of the chromosome (0 Mb) and the distal edge being the one closer to the distal part of the chromosome. In each of the groups V-VII, there are two separate sex-linked regions (“R1” and “R2”) on chromosome 3, and they have therefore one set of NRR edges for each region and group. | | | | | | | | | | | |
| --- | --- | --- | --- | --- | --- | --- | --- | --- | --- | --- | --- |
| **Chromosome** | | **Size of NRR (Mb)** | **Position of NRR edges (Mb)** | | **NRR present (X) or absent (-) in Group** | | | | | | |
| **ID** | **Length (Mb)** |  | **Proximal** | **Distal** | **I** | **II** | **III** | **IV** | **V** | **VI** | **VII** |
| Z | 72.9 | 0.0–72.9 | 0.0 | 72.9 ^#^ | X | X | X | X | X | X | X |
| 4A | 20.7 | 0.0–9.6 | 0.0 | 9.6 ^##^ | - | X | X | - | X | X | X |
| 4A | 20.7 | 5.3–9.6 | 5.3 | 9.6 ^##^ | - | - | - | X | - | - | - |
| 4 | 69.8 | 13.8–49.1 | 13.8 | 49.1 | - | - | X | - | - | - | - |
| 8 | 28.0 | 7.3–21.8 | 7.3 | 21.8 | - | - | - | X | - | - | - |
| 3 | 112.6 | 8.4–10.4 (R1); 18.1–24.1 (R2) | 8.4 (R1); 18.1 (R2) | 10.4 (R1); 24.1 (R2) | - | - | - | - | X | - | - |
| 3 | 112.6 | 8.4–14.0 (R1); 18.1–24.1 (R2) | 8.4 (R1); 18.1 (R2) | 14.0 (R1); 24.1 (R2) | - | - | - | - | - | X | - |
| 3 | 112.6 | 8.4–24.1 (R1); 29.8–88.0 (R2) | 8.4 (R1); 29.8 (R2) | 24.1 (R1); 88.0 (R2) | - | - | - | - | - | - | X |
| 5 | 62.4 | 9.1–45.4 | 9.1 | 45.4 | - | - | - | - | - | - | X |
| *^#, ##^: Previously known fusion point between Z and 4A (distal end of Z, position 72.9 Mb, to distal NRR-end of 4A, position 9.6 Mb).* | | | | | | | | | | | |

| **Supplementary Table 2.** Songbird pseudoautosomal (PAR) genes identified in the *Taeniopygia guttata* annotation (bTaeGut2.pat.W.v2). | | | | | | | | | | | |
| --- | --- | --- | --- | --- | --- | --- | --- | --- | --- | --- | --- |
| **Gene name** | ***Taeniopygia* gene ID** | **Transcript ID** | **Scaffold** | **Start (bp)** | **End (bp)** | **Protein coding** | **Remain-ing after filtering** | **Gene description ^#^** | **Gene ontology: function ^#^** | **Gene ontology: process ^#^** | **Gene ontology: component ^#^** |
| *uncharacterized1* | LOC116806781 | XR_004365834.1 | NC_045027.1 | 11005 | 11912 | no | no | Uncharacterized LOC116806781 |  |  |  |
| *LMAN1* | LOC100220399 | XM_030258642.2 | NC_045027.1 | 33731 | 58078 | yes | yes | Protein ERGIC-53 | D-mannose binding; syntaxin binding | Endoplasmic reticulum to Golgi vesicle-mediated transport; neurotransmitter transport | COPII-coated ER to Golgi transport vesicle; Golgi membrane; endoplasmic reticulum membrane; endoplasmic reticulum-Golgi intermediate compartment |
| *uncharacterized2* | LOC116806731 | XR_004365759.1 | NC_045027.1 | 52211 | 71108 | no | yes | Uncharacterized LOC116806731 |  |  |  |
| *RAX* | RAX | NM_001243734.2 | NC_045027.1 | 64707 | 66358 | yes | yes | Retina and anterior neural fold homeobox | DNA-binding transcription factor activity, RNA polymerase II-specific; RNA polymerase II cis-regulatory region sequence-specific DNA binding | Positive regulation of transcription by RNA polymerase II |  |
| *GRP* | LOC105760884 | XM_030258647.2 | NC_045027.1 | 71706 | 76475 | yes | yes | Gastrin-releasing peptide | Neuropeptide hormone activity | Neuropeptide signaling pathway | Extracellular space |
| *SEC11C* | LOC116806596 | XM_030257196.2 | NC_045027.1 | 79268 | 84487 | yes | yes | Signal peptidase complex catalytic subunit SEC11C |  |  |  |
| *ZNF532* | LOC116806602 | XM_032744080.1 | NC_045027.1 | 90198 | 124057 | yes | yes | Zinc finger protein 532 |  |  |  |
| *MALT1* | LOC116806749 | XM_032744259.1 | NC_045027.1 | 135069 | 153662 | yes | yes | Mucosa-associated lymphoid tissue lymphoma translocation protein 1 homolog |  |  |  |
| *ALPK2* | LOC116806817 | XM_032744489.1 | NC_045027.1 | 153718 | 182131 | yes | yes | Alpha-protein kinase 2-like |  |  |  |
| *uncharacterized3* | LOC116806818 | XR_004365896.1 | NC_045027.1 | 182242 | 185760 | no | yes | Uncharacterized LOC116806818 |  |  |  |
| *MIR122* | MIR122 | NR_049051.1 | NC_045027.1 | 182594 | 182665 | no | no | MicroRNA mir-122 |  |  |  |
| *NEDD4L* | LOC116806603 | XM_032744495.1 | NC_045027.1 | 203780 | 290722 | yes | yes | E3 ubiquitin-protein ligase NEDD4-like | Protein binding; sodium channel inhibitor activity; enables ubiquitin protein ligase activity | Neuromuscular junction development; protein ubiquitination; receptor catabolic process; receptor internalization; regulation of dendrite morphogenesis; ubiquitin-dependent protein catabolic process | Cytoplasm |
| *uncharacterized4* | LOC115491286 | XR_003957248.2 | NC_045027.1 | 209240 | 211463 | no | yes | Uncharacterized LOC115491286 |  |  |  |
| *uncharacterized5* | LOC116806819 | XR_004365897.1 | NC_045027.1 | 215304 | 221401 | no | no | Uncharacterized LOC116806819 |  |  |  |
| *ATP8B1* | LOC116806743 | XM_032744197.1 | NC_045027.1 | 304375 | 325804 | yes | yes | Phospholipid-transporting ATPase IC-like |  |  |  |
| *NARS1* | NARS1 | XM_030259060.2 | NC_045027.1 | 325874 | 333111 | yes | yes | Asparaginyl-tRNA synthetase 1 | ATP binding; asparagine-tRNA ligase activity; enables nucleic acid binding | Asparaginyl-tRNA aminoacylation | Cytoplasm |
| *FECH* | LOC116806605 | XM_030259064.2 | NC_045027.1 | 334740 | 346420 | yes | yes | Ferrochelatase, mitochondrial | Ferrochelatase activity | Heme biosynthetic process | Mitochondrion |
| *ONECUT2* | LOC100226718 | XR_003957244.2 | NC_045027.1 | 350233 | 367344 | yes | no | One cut domain family member 2 |  |  |  |
| *ST8SIA3* | LOC116806742 | XM_032744196.1 | NC_045027.1 | 370724 | 378119 | yes | yes | Sia-alpha-2,3-Gal-beta-1,4-GlcNAc-R:alpha 2,8-sialyltransferase |  |  |  |
| *uncharacterized6* | LOC115491263 | XR_003957222.2 | NC_045027.1 | 379525 | 398137 | no | yes | Uncharacterized LOC115491263 |  |  |  |
| *WDR7* | LOC116806851 | XM_032744613.1 | NC_045027.1 | 400103 | 447553 | yes | yes | WD repeat-containing protein 7-like |  |  |  |
| *TXNL1* | LOC100218989 | XM_032744615.1 | NC_045027.1 | 444330 | 457645 | yes | no | Thioredoxin-like protein 1 |  |  |  |
| *uncharacterized7* | LOC116806852 | XR_004365928.1 | NC_045027.1 | 484714 | 493199 | no | no | Uncharacterized LOC116806852 |  |  |  |
| *^#^ Information from: www.ncbi.nlm.nih.gov/gene (accessed 27 November 2025)* | | | | | | | | | | | |

| **Supplementary Table 3.** Samples and short-read sequences used for analyses. All samples were sequenced by us in previous studies, except for *Ficedula albicollis*. All sequencing data are available on NCBI. | | | | | | |
| --- | --- | --- | --- | --- | --- | --- |
| **Species** | **Common name** | **Female: Sample name** | **Male: Sample name** | **Female: NCBI accession (SRA)** | **Male: NCBI accession (SRA)** | **BioProject** |
| *Aegithalos caudatus* | Long-tailed tit | QF-1504-CP59475_S11_L004 | QF-1504-BL37630_S12_L004 | SRX7707624 | SRX7707625 | PRJNA579268 |
| *Alauda arvensis* | Eurasian skylark | QL-1681-19_S46_L006 | QL-1681-21_S47_L006 | SRX7050286 | SRX7050287 | PRJNA579268 |
| *Cecropis daurica* | Red-rumped swallow | QF-1504-P182141_S2_L001 | QF-1504-P182142_S1_L001 | SRX7707630 | SRX7707631 | PRJNA579268 |
| *Cettia cetti* | Cetti’s warbler | QF-1504-P182137_S9_L003 | QF-1504-2L18122_S4_L002 | SRX7707626 | SRX7707627 | PRJNA579268 |
| *Cisticola juncidis* | Zitting cisticola | QF-1504-CISJUN-2_S6_L002 | QF-1504-RA5680_S5_L002 | SRX7707628 | SRX7707629 | PRJNA579268 |
| *Eremophila alpestris* | Horned lark | QF-1504-H-19_S8_L003 | QF-1504-H-88_S7_L003 | SRX7050288 | SRX7050289 | PRJNA579268 |
| *Locustella luscinioides* | Savi’s warbler | QF-1504-LOCLUS-43_S1_L001 | QF-1504-LOCLUS-24_S3_L001 | SRX7707618 | SRX7707619 | PRJNA579268 |
| *Panurus biarmicus* | Bearded reedling | QF-1504-2KR32024_S2_L001 | QF-1504-1ET92164_S3_L001 | SRX7050290 | SRX7050291 | PRJNA579268 |
| *Phylloscopus collybita* | Common chiffchaff | QF-1504-R86159_S5_L002 | QF-1504-Z81303_S4_L002 | SRX7707620 | SRX7707621 | PRJNA579268 |
| *Sylvia atricapilla* | Eurasian blackcap | 1EL38952_S2_L001 | 1EV02922_S4_L002 | SRX7707622 | SRX7707623 | PRJNA579268 |
| *Acrocephalus schoenobaenus* | Sedge warbler | QF-1504-CT90325_S17_L006 | QF-1504-CT90312_S18_L006 | SRX7707616 | SRX7707617 | PRJNA578893 |
| *Argya altirostris* | Iraq babbler | SJ-2333-IB-2b_S32_L002 | SJ-2333-IB-1a_S31_L002 | SRX16766677 | SRX16766676 | PRJNA578893 |
| *Pycnonotus barbatus* | Common bulbul | SJ-2333-Pbar-197_S24_L002 | SJ-2333-Pbar-421_S22_L002 | SRX16766680 | SRX16766681 | PRJNA578893 |
| *Sylvietta brachyura* | Northern crombec | SJ-2333-Sbra-553_S28_L002 | SJ-2333-Sbra-878_S26_L002 | SRX16766678 | SRX16766679 | PRJNA578893 |
| *Ficedula albicollis* | Collared flycatcher | OC_HB10_450 | OC_1_450 | ERR637378 | ERR637360 | PRJEB7359 |

| **Supplementary Table 4.** Z-to-W branch distances between sexes, per species. | | | | | | | | | | | | | | | | |
| --- | --- | --- | --- | --- | --- | --- | --- | --- | --- | --- | --- | --- | --- | --- | --- | --- |
| **Species** | **No. of genes** | **Z–to–W branch distance** | | | | **Median species > median *Taeniopygia*** | ***W*-statistic ^#^** | ***P*-value** | **Bonferroni corrected *P*-value ^$^** | ***P*-value formatted** | **Bonferroni corrected *P*-value ^$^, formatted** | **Z–W sequence identity (%)** | | | **Read coverage** | |
|  |  | **Median** | **Range** | **SD** | **IQR** |  |  |  |  |  |  | **Mean** | **Range** | **SD** | **Female: Mean ± SD** | **Male: Mean ± SD** |
| *Ficedula albicollis* | 17 | 0.00125 | 0.00000–0.00635 | 0.00174 | 0.00179 | FALSE | 62 | 8.09E-03 | 1.21E-01 | 0.0081 | 0.1213 | 99.84 | 99.38-100.00 | 0.168 | 10.90 ± 4.03 | 14.90 ± 3.62 |
| *Cettia cetti* | 17 | 0.00000 | 0.00000–0.00048 | 0.00019 | 0.00024 | FALSE | 0 | 8.85E-07 | 1.33E-05 | 0.0000 | 0.0000 | 99.99 | 99.95-100.00 | 0.113 | 20.66 ± 1.53 | 23.16 ± 2.55 |
| *Aegithalos caudatus* | 17 | 0.00108 | 0.00000–0.00456 | 0.00136 | 0.00142 | FALSE | 53 | 2.96E-03 | 4.44E-02 | 0.0030 | 0.0444 | 99.85 | 99.57-100.00 | 0.128 | 31.35 ± 1.94 | 26.62 ± 1.46 |
| *Phylloscopus collybita* | 17 | 0.00047 | 0.00000–0.00675 | 0.00255 | 0.00422 | FALSE | 84 | 6.28E-02 | 9.42E-01 | 0.0628 | 0.9421 | 99.79 | 99.36-100.00 | 0.240 | 23.47 ± 2.64 | 30.72 ± 3.03 |
| *Pycnonotus barbatus* | 16 | 0.00096 | 0.00000–0.00363 | 0.00116 | 0.00134 | FALSE | 42 | 7.70E-04 | 1.15E-02 | 0.0008 | 0.0115 | 99.87 | 99.65-100.00 | 0.113 | 43.47 ± 4.28 | 42.43 ± 3.35 |
| *Sylvia atricapilla* | 17 | 0.00569 | 0.00190–0.01057 | 0.00261 | 0.00369 | TRUE | 185 | 8.05E-02 | 1.00E+00 | 0.0805 | 1.0000 | 99.49 | 98.95-99.82 | 0.255 | 37.67 ± 4.26 | 36.01 ± 4.21 |
| *Argya altirostris* | 17 | 0.00000 | 0.00000–0.00243 | 0.00080 | 0.00076 | FALSE | 17 | 1.86E-05 | 2.79E-04 | 0.0000 | 0.0003 | 99.94 | 99.76-100.00 | 0.079 | 27.51 ± 1.88 | 36.76 ± 3.10 |
| *Cecropis daurica* | 17 | 0.00240 | 0.00000–0.00706 | 0.00199 | 0.00203 | FALSE | 96 | 1.55E-01 | 1.00E+00 | 0.1547 | 1.0000 | 99.77 | 99.30-100.00 | 0.195 | 13.62 ± 1.95 | 24.75 ± 2.18 |
| *Acrocephalus schoenobae-nus* | 17 | 0.00381 | 0.00141–0.01432 | 0.00411 | 0.00530 | TRUE | 188 | 6.30E-02 | 9.45E-01 | 0.0630 | 0.9450 | 99.44 | 98.65-99.86 | 0.392 | 21.22 ± 1.95 | 15.84 ± 1.45 |
| *Locustella luscinioides* | 17 | 0.00095 | 0.00000–0.00550 | 0.00149 | 0.00176 | FALSE | 53 | 2.94E-03 | 4.40E-02 | 0.0029 | 0.0440 | 99.86 | 99.49-100.00 | 0.140 | 28.97 ± 2.63 | 42.17 ± 3.02 |
| ***Cisticola juncidis*** | **17** | **0.05870** | **0.03177–0.10206** | **0.01754** | **0.02255** | **TRUE** | **272** | **1.71E-09** | **2.57E-08** | **0.0000** | **0.0000** | **94.83** | **92.14-97.12** | **1.405** | **27.44 ± 2.90** | **28.15 ± 2.34** |
| ***Sylvietta brachyura*** | **17** | **0.02553** | **0.01580–0.03717** | **0.00670** | **0.01014** | **TRUE** | **272** | **1.71E-09** | **2.57E-08** | **0.0000** | **0.0000** | **97.58** | **96.56-98.59** | **0.653** | **27.46 ± 1.93** | **25.48 ± 2.06** |
| *Panurus biarmicus* | 16 | 0.00071 | 0.00000–0.00349 | 0.00105 | 0.00137 | FALSE | 40 | 5.53E-04 | 8.30E-03 | 0.0006 | 0.0083 | 99.89 | 99.66-100.00 | 0.101 | 19.76 ± 2.64 | 31.05 ± 2.09 |
| *Eremophila alpestris* | 17 | 0.00309 | 0.00070–0.00817 | 0.00198 | 0.00184 | TRUE | 143 | 8.17E-01 | 1.00E+00 | 0.8173 | 1.0000 | 99.64 | 99.19-99.93 | 0.192 | 24.42 ± 3.52 | 37.21 ± 3.24 |
| ***Alauda arvensis*** | **16** | **0.01500** | **0.00727–0.02292** | **0.00429** | **0.00387** | **TRUE** | **252** | **3.99E-08** | **5.99E-07** | **0.0000** | **0.0000** | **98.58** | **97.89-99.36** | **0.413** | **34.16 ± 2.51** | **32.50 ± 2.69** |
|  |  |  |  |  |  |  |  |  |  |  |  |  |  |  |  |  |
| *Taeniopygia guttata* | 16 | 0.00243 | 0.00135–0.00889 | 0.00282 | 0.00453 |  |  |  |  |  |  |  |  |  |  |  |
|  |  |  |  |  |  |  |  |  |  |  |  |  |  |  |  |  |
| *^#^ Two-sided Wilcoxon sign-rank tests (species against Taeniopygia)* | | | | | | | | | | | | | | | | |
| *^$^ Bonferroni correction for testing each species (n = 15) against Taeniopygia* | | | | | | | | | | | | | | | | |

| **Supplementary Table 5.** Unique SNP count in males and females for PAR gene. | | | | | | | | | | | | | | | |
| --- | --- | --- | --- | --- | --- | --- | --- | --- | --- | --- | --- | --- | --- | --- | --- |
| **Species** | **No. of genes** | **Number of unique SNPs in females** | | | | **Number of unique SNPs in males** | | | | **Proportion of genes with value higher in female** | ***W*-statistic ^#^** | ***P*-value** | **Bonferroni corrected *P*-value ^$^** | ***P*-value formatted** | **Bonferroni corrected *P*-value ^$^, formatted** |
|  |  | **Median** | **Range** | **SD ^€^** | **IQR ^&^** | **Median** | **Range** | **SD ^€^** | **IQR ^&^** |  |  |  |  |  |  |
| *Ficedula albicollis* | 17 | 2 | 0-17 | 4.07 | 2 | 3 | 0-24 | 5.99 | 4 | 0.12 | 8 | 1.61E-02 | 2.42E-01 | 0.0161 | 0.2416 |
| *Cettia cetti* | 17 | 0 | 0-4 | 1.17 | 1 | 0 | 0-2 | 0.59 | 0 | 0.29 | 24 | 4.30E-01 | 1.00E+00 | 0.4297 | 1.0000 |
| *Aegithalos caudatus* | 17 | 3 | 0-13 | 3.74 | 4 | 2 | 0-12 | 3.59 | 3 | 0.41 | 46 | 1.00E+00 | 1.00E+00 | 1.0000 | 1.0000 |
| *Phylloscopus collybita* | 17 | 1 | 0-17 | 5.16 | 5 | 1 | 0-18 | 5.54 | 8 | 0.18 | 17.5 | 3.29E-01 | 1.00E+00 | 0.3288 | 1.0000 |
| *Pycnonotus barbatus* | 17 | 2 | 0-12 | 3.68 | 3 | 3 | 0-12 | 3.63 | 5 | 0.41 | 51 | 6.27E-01 | 1.00E+00 | 0.6271 | 1.0000 |
| *Sylvia atricapilla* | 17 | 8 | 0-30 | 8.47 | 11 | 10 | 0-21 | 5.89 | 8 | 0.59 | 81.5 | 5.01E-01 | 1.00E+00 | 0.5007 | 1.0000 |
| *Argya altirostris* | 17 | 1 | 0-37 | 8.80 | 3 | 0 | 0-5 | 1.23 | 1 | 0.59 | 65.5 | 3.98E-02 | 5.97E-01 | 0.0398 | 0.5975 |
| *Cecropis daurica* | 17 | 4 | 0-16 | 4.84 | 6 | 3 | 0-12 | 4.10 | 5 | 0.53 | 73 | 4.76E-01 | 1.00E+00 | 0.4760 | 1.0000 |
| *Locustella luscinioides* | 17 | 1 | 0-36 | 8.96 | 5 | 1 | 0-11 | 3.08 | 1 | 0.41 | 52 | 6.73E-01 | 1.00E+00 | 0.6726 | 1.0000 |
| *Acrocephalus schoenobaenus* | 17 | 11 | 0-40 | 11.41 | 15 | 9 | 1-32 | 9.01 | 11 | 0.59 | 87.5 | 1.24E-01 | 1.00E+00 | 0.1239 | 1.0000 |
| ***Cisticola juncidis*** | **17** | **125** | **31-386** | **96.55** | **103** | **0** | **0-0** | **0.00** | **0** | **1.00** | **153** | **3.20E-04** | **4.81E-03** | **0.0003** | **0.0048** |
| ***Sylvietta brachyura*** | **17** | **62** | **13-196** | **49.14** | **51** | **1** | **0-7** | **1.94** | **2** | **1.00** | **153** | **3.20E-04** | **4.81E-03** | **0.0003** | **0.0048** |
| *Panurus biarmicus* | 17 | 2 | 0-13 | 3.27 | 3 | 1 | 0-9 | 2.65 | 4 | 0.53 | 71 | 5.45E-01 | 1.00E+00 | 0.5455 | 1.0000 |
| *Eremophila alpestris* | 17 | 5 | 0-58 | 13.81 | 9 | 4 | 0-15 | 4.45 | 5 | 0.53 | 90.5 | 2.54E-01 | 1.00E+00 | 0.2540 | 1.0000 |
| ***Alauda arvensis*** | **17** | **32** | **0-99** | **25.10** | **32** | **5** | **0-32** | **9.54** | **12** | **0.94** | **136** | **4.81E-04** | **7.22E-03** | **0.0005** | **0.0072** |
| *^#^ Two-sided paired Wilcoxon sign-rank tests (males against females)* | | | | | | | | | | | | | | | |
| *^$^ Bonferroni correction for testing all 15 species* | | | | | | | | | | | | | | | |
| *^€^ Standard deviation* | | | | | | | | | | | | | | | |
| *^&^ Interquartile range* | | | | | | | | | | | | | | | |

| **Supplementary Table 6.** Published reference genomes used in the study to identify fusion points and novel PARs, and what samples (from Supplementary Table 3) were aligned to each reference genome (see Methods). | | | | | | |
| --- | --- | --- | --- | --- | --- | --- |
| **Species** | **Common name** | **Reference genome (downloaded)** | | | **Analysed with (Supplementary Table 3)** | |
|  |  | **Assembly name** | **NCBI assembly accession** | **Sex of assembly individual** | **Female** | **Male** |
| *Alauda arvensis* | Eurasian skylark | skylark_genome | GCA_902810485.1 | male | QL-1681-19_S46_L006 | QL-1681-21_S47_L006 |
| *Alauda arvensis* | Eurasian skylark | skylark_genome | GCA_902810485.1 | male | QL-1681-95694_S52_L008 | QL-1681-246_S51_L008 |
| *Cisticola juncidis* | Zitting cisticola | ASM1340021v1 | GCA_013400215.1 | male | QF-1504-CISJUN-2_S6_L002 | QF-1504-RA5680_S5_L002 |
| *Eremophila alpestris* | Horned lark | CLO_EAlp_1.0 | GCA_009792885.1 | male | QF-1504-H-19_S8_L003 | QF-1504-H-88_S7_L003 |
| *Sylvietta virens* | Green crombec | ASM1339951v1 | GCA_013399515.1 | male | SJ-2333-Sbra-553_S28_L002 | SJ-2333-Sbra-878_S26_L002 |

| **Supplementary Table 7.** Scaffolds suggestive of fusion points between chromosome 4A and chromosome 3. | | | | | |
| --- | --- | --- | --- | --- | --- |
| **Species** | **Description** | **Scaffold ID** | **Scaffold range (bp)** | ***Taeniopygia* range (bp)** | **Sex-linked** |
| *Eremophila alpestris* | Fusion scaffold (Z;4A;3) | WMCF01000024.1 | 0-3269374 | Z:69365659-72860660 | sex-linked |
| *Eremophila alpestris* | Fusion scaffold (Z;4A;3) | WMCF01000024.1 | 3277173-12546504 | 4A:4588-9605363 | sex-linked |
| *Eremophila alpestris* | Fusion scaffold (Z;4A;3) | WMCF01000024.1 | 12550475-12555560 | 3:12208-18513 | sex-linked |
| *Eremophila alpestris* | Fusion scaffold (Z;4A;3) | WMCF01000024.1 | 12558560-12560541 | 3:24098751-24103322 | sex-linked |
| *Alauda arvensis* | Fusion scaffold (4A;3) | CADDXX010000006.1 | 15000-5830000 | 4A:23654-5995271 | sex-linked |
| *Alauda arvensis* | Fusion scaffold (4A;3) | CADDXX010000006.1 | 5000-15000 | 3:14664-16406 | unsure |
| *Alauda arvensis* | Fusion scaffold (4A;3) | CADDXX010000006.1 | 0-5000 | 3:24099520-24099841 | sex-linked |

| **Supplementary Table 8.** Suggested neo-Z chromosome structure for Groups II-VII (see Figure 3). | |
| --- | --- |
| **Group** | **Z chromosome structure** |
| II | PARZZ:0.0-0.5(+)-NRRZZ:0.5-73.4(+)-NRR4A4A:9.6-0.0(-) |
| III | PAR44:69.8-49.1(-)-NRR44:49.1-13.8(-)-NRRZZ:0.0-73.4(+)-NRR4A4A:9.6-0.0(-) |
| IV | PAR88:28.0-21.8(-)-NRR88:21.8-7.3(-)-NRRZZ:0.0-73.4(+)-NRR4A4A:9.6-5.3(-)-PAR4A4A:5.3-00(-) |
| V | PARZZ:0.0-0.5(+)-NRRZZ:0.5-73.4(+)-NRR4A4A:9.6-0.0(-)-NRR33:unsure-PAR33:unsure, |
| VI | PAR55:62.4-0.0(-)-PARZZ:0.0-0.5(+)-NRRZZ:0.5-73.4(+)-NRR4A4A:9.6-0.0(-)-NRR33:unsure-PAR33:unsure |
| VII | PAR55:62.4-45.4(-)-NRR55:45.1-9.1(-)-NRRZZ:0.0-73.4(+)-NRR4A4A:9.6-0.0(-)-NRR33:unsure-PAR33:unsure |

| **Supplementary Table 9.** Songbird PAR-scaffolds from reference genomes (in Supplementary Table 6) used to generate Supplementary Figure 10. | | |
| --- | --- | --- |
| **Species** | **Chromosome copy** | **ID** |
| *Taeniopygia guttata* (3.2.4) | PAR-Z | Z_random |
| *Taeniopygia guttata* (bTaeGut2.pat.W.v2) | PAR-Z | NC_045027.1 |
| *Taeniopygia guttata* (bTaeGut2.pat.W.v2) | PAR-W | NW_022611471.1 |
| *Ficedula albicollis* | PAR-Z | N00298 |
| *Ficedula albicollis* | PAR-Z | N00378 |
| *Ficedula albicollis* | PAR-Z | N02597 |
| *Sylvietta virens* | PAR-Z | scaffold_394 |
| *Alauda arvensis* | PAR-Z | CADDXX010000025.1 |

| **Supplementary Table 10.** Spearman rank statistics (values also given in Figure 4 and Supplementary Figure 12). | | | | | |
| --- | --- | --- | --- | --- | --- |
| **Species** | ***Rho*** | **Number of genes** | ***P*-value** | ***P*-value, formatted** | **Figure** |
| *Acrocephalus schoenobaenus* (Group II: Z, 4A) | 0.713 | 17 | 1.31E-03 | **0.001** | Fig. 4 |
| *Aegithalos caudatus* (Group II: Z, 4A) | 0.692 | 17 | 2.10E-03 | **0.002** | Fig. 4 |
| *Argya altirostris* (Group II: Z, 4A) | -0.085 | 17 | 7.45E-01 | 0.745 | Fig. 4 |
| *Cecropis daurica* (Group II: Z, 4A) | 0.644 | 17 | 5.29E-03 | **0.005** | Fig. 4 |
| *Cettia cetti* (Group II: Z, 4A) | 0.076 | 17 | 7.71E-01 | 0.771 | Fig. 4 |
| *Eremophila alpestris* (Group VI: Z, 4A, 3, (5)) | 0.348 | 17 | 1.71E-01 | 0.171 | Fig. 4 |
| *Ficedula albicollis* (Group I: Z) | -0.101 | 17 | 7.00E-01 | 0.700 | Fig. 4 |
| *Locustella luscinioides* (Group II: Z, 4A) | 0.554 | 17 | 2.11E-02 | **0.021** | Fig. 4 |
| *Panurus biarmicus* (Group V: Z, 4A, 3, 5) | 0.518 | 16 | 4.00E-02 | **0.040** | Fig. 4 |
| *Phylloscopus collybita* (Group II: Z, 4A) | 0.529 | 17 | 2.91E-02 | **0.029** | Fig. 4 |
| *Taeniopygia guttata* (Group I: Z) | 0.838 | 16 | 5.01E-05 | **0.000** | Fig. 4 |
| *Alauda arvensis* (Group VII: Z, 4A, 3, 5) | 0.315 | 16 | 2.35E-01 | 0.235 | Suppl. Fig. 12 |
| *Cisticola juncidis* (Group III: Z, 4A, 4) | 0.167 | 17 | 5.23E-01 | 0.523 | Suppl. Fig. 12 |
| *Pycnonotus barbatus* (Group II: Z, 4A) | 0.274 | 16 | 3.05E-01 | 0.305 | Suppl. Fig. 12 |
| *Sylvia atricapilla* (Group II: Z, 4A) | -0.245 | 17 | 3.43E-01 | 0.343 | Suppl. Fig. 12 |
| *Sylvietta brachyura* (Group IV: Z, 4A, 8) | 0.333 | 17 | 1.91E-01 | 0.191 | Suppl. Fig. 12 |

**Supplementary References**

[1] Ankenbrand MJ, Hohlfeld S, Hackl T, Förster F. (2017) AliTV—interactive visualization of

whole genome comparisons. *PeerJ Computer Science* **3**:e116.
