# Supplementary material for: Evolutionary dynamics of enlarged neo-sex chromosomes and novel pseudoautosomal regions in Sylvioidea songbirds
Source: Commun Biol. 2026 Jul 4;9:898. doi: 10.1038/s42003-026-10603-3 (PMC13333021; doi:10.1038/s42003-026-10603-3)
Supplement: Supplementary file 4 — Reporting Summary [file 42003_2026_10603_MOESM4_ESM.pdf]

nature portfolio

Corresponding author(s): Bengt Hansson

Last updated by author(s): May 26, 2026

Reporting Summary

Nature Portfolio wishes to improve the reproducibility of the work that we publish. This form provides structure for consistency and transparency in reporting. For further information on Nature Portfolio policies, see our Editorial Policies and the Editorial Policy Checklist.

Statistics

For all statistical analyses, confirm that the following items are present in the figure legend, table legend, main text, or Methods section.

n/a	Confirmed	
☐	☒	The exact sample size (*n*) for each experimental group/condition, given as a discrete number and unit of measurement
☒	☐	A statement on whether measurements were taken from distinct samples or whether the same sample was measured repeatedly
☐	☒	The statistical test(s) used AND whether they are one- or two-sided<br>*Only common tests should be described solely by name; describe more complex techniques in the Methods section.*
☒	☐	A description of all covariates tested
☐	☒	A description of any assumptions or corrections, such as tests of normality and adjustment for multiple comparisons
☐	☒	A full description of the statistical parameters including central tendency (e.g. means) or other basic estimates (e.g. regression coefficient) AND variation (e.g. standard deviation) or associated estimates of uncertainty (e.g. confidence intervals)
☐	☒	For null hypothesis testing, the test statistic (e.g. *F*, *t*, *r*) with confidence intervals, effect sizes, degrees of freedom and *P* value noted<br>*Give P values as exact values whenever suitable.*
☒	☐	For Bayesian analysis, information on the choice of priors and Markov chain Monte Carlo settings
☒	☐	For hierarchical and complex designs, identification of the appropriate level for tests and full reporting of outcomes
☒	☐	Estimates of effect sizes (e.g. Cohen's *d*, Pearson's *r*), indicating how they were calculated

Our web collection on statistics for biologists contains articles on many of the points above.

Software and code

Policy information about availability of computer code

Data collection	We have only used already published bioinformatics software. We provide all code/scripts as supplementary material.
Data analysis	We have only used already published bioinformatics software. We provide all code/scripts as supplementary material.

For manuscripts utilizing custom algorithms or software that are central to the research but not yet described in published literature, software must be made available to editors and reviewers. We strongly encourage code deposition in a community repository (e.g. GitHub). See the Nature Portfolio guidelines for submitting code & software for further information.

Data

Policy information about availability of data

All manuscripts must include a data availability statement. This statement should provide the following information, where applicable:

- Accession codes, unique identifiers, or web links for publicly available datasets
- A description of any restrictions on data availability
- For clinical datasets or third party data, please ensure that the statement adheres to our policy

All data we have used in the MS are uploaded and available at NCBI (see Supplementary Tables 3 and 6).

Research involving human participants, their data, or biological material

Policy information about studies with human participants or human data. See also policy information about sex, gender (identity/presentation), and sexual orientation and race, ethnicity and racism.

Reporting on sex and gender	*Use the terms sex (biological attribute) and gender (shaped by social and cultural circumstances) carefully in order to avoid confusing both terms. Indicate if findings apply to only one sex or gender; describe whether sex and gender were considered in study design; whether sex and/or gender was determined based on self-reporting or assigned and methods used. Provide in the source data disaggregated sex and gender data, where this information has been collected, and if consent has been obtained for sharing of individual-level data; provide overall numbers in this Reporting Summary. Please state if this information has not been collected. Report sex- and gender-based analyses where performed, justify reasons for lack of sex- and gender-based analysis.*
Reporting on race, ethnicity, or other socially relevant groupings	*Please specify the socially constructed or socially relevant categorization variable(s) used in your manuscript and explain why they were used. Please note that such variables should not be used as proxies for other socially constructed/relevant variables (for example, race or ethnicity should not be used as a proxy for socioeconomic status). Provide clear definitions of the relevant terms used, how they were provided (by the participants/respondents, the researchers, or third parties), and the method(s) used to classify people into the different categories (e.g. self-report, census or administrative data, social media data, etc.) Please provide details about how you controlled for confounding variables in your analyses.*
Population characteristics	*Describe the covariate-relevant population characteristics of the human research participants (e.g. age, genotypic information, past and current diagnosis and treatment categories). If you filled out the behavioural & social sciences study design questions and have nothing to add here, write "See above."*
Recruitment	*Describe how participants were recruited. Outline any potential self-selection bias or other biases that may be present and how these are likely to impact results.*
Ethics oversight	*Identify the organization(s) that approved the study protocol.*

Note that full information on the approval of the study protocol must also be provided in the manuscript.

Field-specific reporting

Please select the one below that is the best fit for your research. If you are not sure, read the appropriate sections before making your selection.

☐ Life sciences ☐ Behavioural & social sciences ☒ Ecological, evolutionary & environmental sciences

For a reference copy of the document with all sections, see nature.com/documents/nr-reporting-summary-flat.pdf

Ecological, evolutionary & environmental sciences study design

All studies must disclose on these points even when the disclosure is negative.

Study description	We study recombination suppression and its consequences on the W chromosome by analysing sequence variation between males and females at genes located on sex chromosomes in Sylvioidea passerine birds. Sylvioidea species have enlarged sex chromosomes due to translocations of autosomal chromosome regions. Different lineages within Sylvioidea have different translocations, and some translocations are unique to specific lineages. This study system turned out to be particularly informative for understanding the evolutionary dynamics of the pseudoautosomal region (PAR), which is the focus of the MS.
Research sample	We analyse paired-end whole-genome sequence data of males and females of 14 Sylvioidea species and two outgroup species (flycatcher and zebra finch). We have also analysed already published genome assembly data of selected species. Information about the species, samples and NCBI accession numbers is provided in Suppl. Table S3 and S6.
Sampling strategy	Sylvioidea species have enlarged sex chromosomes due to translocations of autosomal chromosome regions. Different lineages within Sylvioidea have different translocations, and some translocations are unique to specific lineages. Our strategy was to include and analyse species representatives of lineages with different translocations.
Data collection	We have used whole-genome sequence data, Illumina paired-end short-read data, of male and female Sylvioidea species. This data were generated by us previously and everything is already uploaded at NCBI. We have also used and analysed publicly available genome assemblies of a few Sylvioidea species (data generated by other researchers, and available at NCBI). All samples, data and NCBI accession numbers are given in Table S3 and S6.
Timing and spatial scale	Not relevant as our samples are modern, whereas we have studied genomic processes over phylogenetic time spans.
Data exclusions	We identified 23 PAR genes of which 17 remained after alignment and filtering of short sequences (<500 bp). Of the 17 PAR genes analysed, almost all genes were analysed in all species (due to too short alignments 1 gene was removed from the analyses in two species, and 2 genes were removed in one species).
Reproducibility	We provide all code/scripts to ensure reproducibility.

Randomization	This study compares males and females and has no randomization.
Blinding	We selected species to represent different lineages within Sylvioidea, and thus blinding is not applicable.

Did the study involve field work? ☐ Yes ☒ No

Reporting for specific materials, systems and methods

We require information from authors about some types of materials, experimental systems and methods used in many studies. Here, indicate whether each material, system or method listed is relevant to your study. If you are not sure if a list item applies to your research, read the appropriate section before selecting a response.

Materials & experimental systems

n/a	Involved in the study
☒	☐ Antibodies
☒	☐ Eukaryotic cell lines
☒	☐ Palaeontology and archaeology
☐	☒ Animals and other organisms
☒	☐ Clinical data
☒	☐ Dual use research of concern
☒	☐ Plants

Methods

n/a	Involved in the study
☒	☐ ChIP-seq
☒	☐ Flow cytometry
☒	☐ MRI-based neuroimaging

Animals and other research organisms

Policy information about studies involving animals; ARRIVE guidelines recommended for reporting animal research, and Sex and Gender in Research

Laboratory animals	NA
Wild animals	We have used sequence data publicly available at NCBI. Short-read data of Sylvioidea species had been generated by us and used in previous publications. DNA was extracted from a blood sample of wild captured individuals, which were released after examination. Sampling was conduced with permission from the Malmö/Lund Ethical Committee for scientific work on animals, Sweden, no. 17277-18.
Reporting on sex	Sex of each sample is provided in Supplementary Table 3.
Field-collected samples	See above ("Wild animals").
Ethics oversight	See above ("Wild animals").

Note that full information on the approval of the study protocol must also be provided in the manuscript.

Plants

Seed stocks	*Report on the source of all seed stocks or other plant material used. If applicable, state the seed stock centre and catalogue number. If plant specimens were collected from the field, describe the collection location, date and sampling procedures.*
Novel plant genotypes	*Describe the methods by which all novel plant genotypes were produced. This includes those generated by transgenic approaches, gene editing, chemical/radiation-based mutagenesis and hybridization. For transgenic lines, describe the transformation method, the number of independent lines analyzed and the generation upon which experiments were performed. For gene-edited lines, describe the editor used, the endogenous sequence targeted for editing, the targeting guide RNA sequence (if applicable) and how the editor was applied.*
Authentication	*Describe any authentication procedures for each seed stock used or novel genotype generated. Describe any experiments used to assess the effect of a mutation and, where applicable, how potential secondary effects (e.g. second site T-DNA insertions, mosiacism, off-target gene editing) were examined.*
